# Semaglutide reduces alcohol intake and relapse-like drinking in male and female rats

**DOI:** 10.1016/j.ebiom.2023.104642

**Published:** 2023-06-07

**Authors:** Cajsa Aranäs, Christian E. Edvardsson, Olesya T. Shevchouk, Qian Zhang, Sarah Witley, Sebastian Blid Sköldheden, Lindsay Zentveld, Daniel Vallöf, Maximilian Tufvesson-Alm, Elisabet Jerlhag

**Affiliations:** Department of Pharmacology, Institute of Neuroscience and Physiology, The Sahlgrenska Academy at the University of Gothenburg, Gothenburg, Sweden

**Keywords:** GLP-1, Nucleus accumbens, Gut-brain axis, Addition, Dopamine, Reward

## Abstract

**Background:**

Glucagon-like peptide1 receptor (GLP-1R) agonists have been found to reduce alcohol drinking in rodents and overweight patients with alcohol use disorder (AUD). However, the probability of low semaglutide doses, an agonist with higher potency and affinity for GLP-1R, to attenuate alcohol-related responses in rodents and the underlying neuronal mechanisms is unknown.

**Methods:**

In the intermittent access model, we examined the ability of semaglutide to decrease alcohol intake and block relapse-like drinking, as well as imaging the binding of fluorescently marked semaglutide to nucleus accumbens (NAc) in both male and female rats. The suppressive effect of semaglutide on alcohol-induced locomotor stimulation and *in vivo* dopamine release in NAc was tested in male mice. We evaluated effect of semaglutide on the *in vivo* release of dopamine metabolites (DOPAC and HVA) and gene expression of enzymes metabolising dopamine (MAOA and COMT) in male mice.

**Findings:**

In male and female rats, acute and repeated semaglutide administration reduced alcohol intake and prevented relapse-like drinking. Moreover, fluorescently labelled semaglutide was detected in NAc of alcohol-drinking male and female rats. Further, semaglutide attenuated the ability of alcohol to cause hyperlocomotion and to elevate dopamine in NAc in male mice. As further shown in male mice, semaglutide enhanced DOPAC and HVA in NAc when alcohol was onboard and increased the gene expression of COMT and MAOA.

**Interpretation:**

Altogether, this indicates that semaglutide reduces alcohol drinking behaviours, possibly via a reduction in alcohol-induced reward and NAc dependent mechanisms. As semaglutide also decreased body weight of alcohol-drinking rats of both sexes, upcoming clinical studies should test the plausibility that semaglutide reduces alcohol intake and body weight in overweight AUD patients.

**Funding:**

10.13039/501100004359Swedish Research Council (2019-01676), LUA/ALF (723941) from the 10.13039/501100005754Sahlgrenska University Hospital and the Swedish brain foundation.

**Research in context**

**Evidence before this study**

The glucagon-like peptide1 receptor (GLP-1R) has recently emerged as a viable candidate target to treat alcohol use disorder (AUD). Specifically, agonists of the GLP-1R have previously been shown to prevent alcohol-related responses in rodents and reduce alcohol drinking in overweight patients with AUD. Semaglutide is the first long-acting and orally-administered GLP-1R agonist that has been clinically approved for the treatment of type II diabetes and obesity. Moreover, semaglutide has higher potency and affinity for the GLP-1R. While a high dose of semaglutide (0.1 mg/kg) acutely decreases alcohol intake in male rats, the influence of low semaglutide doses (0.026 and 0.052 mg/kg) on alcohol-related responses tentatively associated with AUD processes are unknown.

**Added value of this study**

This study provides insight into semaglutide's ability to reduce alcohol intake, prevent relapse drinking in alcohol-drinking male and female rats. Moreover, it further reveales that semaglutide prevents alcohol's stimulatory and dopamine-enhancing properties. It is further found that semaglutide together with alcohol enhances the dopamine metabolism within the nucleus accumbens and that semaglutide binds to this area in alcohol-drinking rats of both sexes.

**Implications of all the available evidence**

This study provides evidence for the anecdotal reports of obese patients treated with semaglutide who also claim reduced alcohol intake. Hence, future research should explore the possibility of semaglutide decreasing alcohol intake in patients with AUD, particularly those who are overweight.

## Introduction

Alcohol use disorder (AUD) is a prevalent neuropsychiatric disorder, considered as a major health and socio-economic burden for individuals and society at large.^1^ It is a multifaceted condition where high intake over prolonged period of time is important for the manifestation of the disease. Moreover, relapse drinking is another major challenge for patients with AUD. Both alcohol drinking and relapse drinking are to some extent driven by alcohol's rewarding properties (for review see^2^), which in humans correlates with an increase in dopamine in nucleus accumbens (NAc).^3^ This dopamine elevation is also evident in rodents and further associated with a locomotor stimulation in male mice.^4^, ^5^, ^6^, ^7^, ^8^ Together these behaviours reflect activation of the mesolimbic dopamine system, and tentatively reward-like behaviours (for review see^9^). While sensitivity to alcohol's rewarding properties is regarded as a risk factor for the development of AUD later in life,^10^ additional factors exist. AUD is a multifaceted disorder, and it involves several crucial aspects such as stress reactivity, impulsivity, decision-making, emotional regulation, and social factors. Thus, these processes need to be taken into account for the treatment and prevention of this disorder.

Given the heterogeneity of AUD, the few available treatment options display limited clinical efficacy.^11^ The need for new therapies is thus substantial and novel treatments can be identified by establishing the neurobiological mechanisms underlying the development of AUD. Although these neurobiological mechanisms are complex, both preclinical and clinical findings suggest the gut-brain peptide glucagon-like peptide-1 (GLP-1) as an important modulator of the neurobiological mechanisms underlying the development of AUD.^12^, ^13^, ^14^ Specifically, several GLP-1 receptor (GLP-1R) agonists suppress alcohol intake, alcohol-seeking behaviour and alcohol-induced activation of the mesolimbic dopamine system in male rodents,^15^, ^16^, ^17^, ^18^ a finding replicated in female rats.^17^ Similarly, the GLP-1R agonist exenatide reduces alcohol consumption in overweight AUD patients^19^ and polymorphisms of the GLP-1R is associated with AUD diagnosis in humans.^14^ Besides establishing that GLP-1 pathway may serve as potential treatment of AUD, these data provide validity to the preclinical studies. Although numerous GLP-1R agonists are available clinically, semaglutide is the first long-acting agent used perorally for both diabetes type II^20^ and obesity.^21^ Compared to other GLP-1R agonists, semaglutide has a greater weight loss and glucose-lowering effect, which is attributed to its higher potency and affinity for GLP-1R (for review see^22^). Due to adverse effects associated with its clinical use, the implementation of lower doses of semaglutide may confer potential benefits. While a high dose of semaglutide has been shown to acutely decrease alcohol intake in male rats,^23^ the effects of lower doses of semaglutide on alcohol-related responses potentially associated with AUD have yet to be defined. Additionally, the underlying neurobiological mechanisms of semaglutide remains unknown. Therefore, we conducted a series of experiments combining behavioural, neurochemical, and molecular approaches in rats and mice to investigate these knowledge gaps.

Initial tests of alcohol-drinking male and female rats explored the ability of acute or repeated administration of low semaglutide doses to reduce alcohol consumption. Further experiments were conducted on alcohol-drinking rats of both sexes to evaluate the potential of semaglutide in preventing relapse-like drinking following alcohol abstinence. As alcohol drinking and relapse-like drinking involve reward-like behaviours, additional tests were conducted in male mice to explore the potential impact of semaglutide on such behaviours. Therefore, the efficacy of semaglutide to inhibit alcohol-induced hyperlocomotion and lower the dopamine levels in NAc shell (NAcS) was investigated. On a similar note, the possibility that semaglutide suppress the intake of rewarding/palatable foods was tested. As dopamine signalling in NAc participates in alcohol's reward-associated responses we explore the potential interaction between semaglutide and dopamine signalling in NAc in a series of experiment. After systemic injection of semaglutide, we imaged the binding of fluorescently labelled semaglutide in the NAcS of alcohol-drinking male and female rats. Then, we investigated the possibility that semaglutide augments the levels of dopamine metabolites and the expression of enzymes degrading dopamine in NAc in male mice with alcohol onboard. To confirm this effect, the *ex vivo* levels of dopamine and its metabolites were measured in NAc in alcohol-drinking male and female rats treated repeatedly with semaglutide. To further explore the interaction between semaglutide and dopamine, semagltuide's suppressive effect on dopamine driven behaviours^24^^,^^25^ was examined in alcohol-naïve male and female rats.

Together, the present behavioural, neurochemical and molecular experiments provide further insight into the potential of semaglutide to reduce several alcohol-related responses tentatively via dopamine mechanisms within NAc. These data may provide a basis for future clinical trials testing semaglutide treatment in patients with AUD.

## Methods

### Animals

Adult age-matched male and female Rcc/Han Wistar rats (weight at arrival was approximately 150–200 g for females, and 200–240 g for males; Envigo, Horst, Netherlands) were used as this species and strain evinces a voluntary high and stable alcohol consumption inducing pharmacologically relevant blood alcohol concentrations.^26^ Rcc/Han Wistar rats were chosen since prior research shows that other GLP-1R agonists decreases alcohol consumption in the same strain^15^, ^16^, ^17^ and as mice in our hands display less robust alcohol intake. Although mice were not used for alcohol intake studies, a similar outcome is expected in mice as previous studies show a similar treatment effect by other GLP-1R agonists on alcohol intake in rats, mice and non-human primates.^16^^,^^18^^,^^27^, ^28^, ^29^ Adult male NMRI mice (approximately 28–32 g at arrival; Charles River, Sulzfeld, Germany) were used when assessing the treatment response on alcohol-induced locomotor stimulation, dopamine release in NAc and conditioned place preference (CPP). In contrast to rats, male NMRI mice were used since this sex and strain previously displayed a distinct stimulatory response to alcohol. This allows for comparison between previously acquired data where male mice have been used.^30^^,^^31^ It is imperative to take into account the potential differences in response and molecular mechanisms between sexes, as female mice were omitted for the mice experiments. Although it might be beneficial to conduct all experiments in one species, rats or mice were chosen to the test where the most robust outcome is obtained. Moreover, the combination of data from rats and mice is commonly observed as they reflect different aspects of alcohol responses.^15^^,^^16^^,^^30^^,^^32^^,^^33^

For all experiments, both rats and mice had free access to tap water and food (chow) and were kept in rooms with 20 °C and 50% humidity. All animals had standardised enrichment according to protocol at the animal facility. Rats in the alcohol drinking experiment were maintained at a reversed light/dark cycle, while a regular 12/12-h light/dark cycle was used for the rodents in the other experiments. Before any test, the rodents habituated at least one week in the animal facility and 1 h to the experimental room.

### Ethics

To adhere to the principles of the 3R (refine, reduce, replace), we limit the number of animals used in each treatment group based on previous experience and prior power analysis. The sample size was determined through a power analysis based on a 5% significance level, an effect size of 0.2 standard deviations or higher, a two-tailed direction of the effect and an 80% study power. We consider a sample size of seven or more sufficient to demonstrate statistically significant effects. Our general pre-set exclusion criteria eliminate rodents with abnormal behaviours, poor health or weight loss exceeding 15%. Specific exclusion criteria for each test are define under each experimental section. We also follow the ARRIVE (Animal Research: Reporting of In Vivo Experiments) guidelines and obtained ethical approval for each experiment from the Swedish Ethical Committee on Animal Research in Gothenburg, Sweden (ethical numbers: 1457/18, 3276/18, 3348/20). All experiments adhered animal use guidelines and ethical approval. Endpoints according to approved ethics is substantial weight loss, alterations of normal behaviours or if the surgically implanted probes were displaced from skull bone. Besides the exploration and novelty seeking experiment, no animal was included in more than one test.

### Drugs

For the drinking experiments alcohol (95%; Solveco, Stockholm, Sweden) was diluted in tap water to a final concentration of 20%. For experiments using systemic administration, 15% alcohol (1.75 g/kg) was dissolved in vehicle (0.9% NaCl) and was injected intraperitoneally 5 min prior to the experiment. Semaglutide (Apoteket AB, Gothenburg, Sweden) was dissolved in vehicle (0.9% NaCl) and injected subcutaneously (sc) 60 min prior to alcohol exposure or behavioural experiment. Semaglutide was obtained as a solution ready for human use, extensively dissolved before injection, and therefore, no additional substances were added to the vehicle. It was used at two doses (0.026 or 0.052 mg/kg), which are lower than the dose tested before (0.1 mg/kg).^23^ As the initial drinking experiment revealed that the lowest dose (0.026 mg/kg) reduced alcohol intake, this dose was used for subsequent experiments. Additionally, the higher dose was avoided for further use as it in male rats lowered the water intake. Although semaglutide is administered weekly in humans it has to be injected daily in rodents due to its rapid metabolism in rodents.^34^ The fluorescently labelled semaglutide (CY3-semaglutide; NNC0113-0217; NovoNordisk Global, Bagsværd, Denmark; 0.026 mg/kg active peptide; sc) was dissolved in vehicle (7.9 ml of 50 mM sodium phostphate, 70 mM sodium chloride and 0.007% polysorbate 20 in MQ water) as instructed by NovoNordisk.^35^

### Experimental designs

As summarised in Table 1, a combination of behavioural, neurochemical and molecular experiments have been used to highlight the ability of semaglutide to reduce alcohol-related responses and to define molecular underpinnings. Before each experiment was conducted, a protocol describing the research question, key design features, and analysis plan was prepared. The experimenter who performed the experiment, collected data and did the initial analysis was aware of the treatment allocation. To limit the possible confounding factor for this, only pre-set exclusion criteria was used when analysing the data obtained. Additionally, the principal investigator unaware of treatments did a final check of all raw data and statistical analysis. To minimise the influence of potential confounders, the order of treatments was stratified, with treatments being mixed for each animal cage and for each apparatus used in the experiment.

**Table 1:** Summary of experiments conducted.

No	Experiment	Species	Sex	Rational	Main figure	Additional outcome
1	Effects of semaglutide on alcohol intake for 8–10 weeks a. Acute, 0.026 mg/kg b. Acute, 0.052 mg/kg c. Repeated, 0.026 mg/kg	Rats	Males Females	High intake over prolonged period of time is important for the manifestation of alcohol use disorder.	1	Influence of semaglutide on food intake (Fig. 2), alcohol preference, water and total fluid intake and body weight (Supplementary Figs. S2, S3 and S5)
2	Effects of semaglutide on relapse-like drinking in the alcohol deprivation model	Rats	Males Females	Abstinence to alcohol causes relapse-like drinking, a behaviour reflecting the inability to abstain from alcohol. This is an important aspect of alcohol use disorder.	1	Influence of semaglutide on alcohol preference, water, total fluid and food intake as well as body weight (Supplementary Fig. S7)
3	Effects of semaglutide treatment on alcohol-induced locomotor stimulation	Mice	Males	Alcohol causes a release of dopamine in NAcS in rodents and humans, an effect associated with an increase locomotor activity, thus reflecting the acute effects of alcohol.	3	Effects on other locomoto activity parameters (Supplementary Fig. S8)
4	Effects of semaglutide treatment on alcohol-induced dopamine release in NAcS, *in vivo*	Mice	Males	The ability of alcohol to enhance dopamine in NAcS is associated with the rewarding experience in man and is a risk factor for a later alcohol use disorder diagnosis	3	Effects of semaglutide together with alcohol on the release of dopamine metabolites (HVA, DOPAC; Fig. 4). These additional data explore the possibility that semaglutide together with alcohol changes the metabolism of dopamine. This contributes to the identification of underlying molecular underpinnings. Effects on other monoaminergic neurotransmission in NAcS (Supplementary Fig. S11).
5	Effects of semaglutide treatment on alcohol-induced conditioned place preference	Mice	Males	The memory of the alcohol reward is an important aspect of the alcohol use disorder process, a behaviour that can be measured in the memory of conditioned place preference test	Result section	
6	Effects of semaglutide treatment on the intake of rewarding foods	Mice	Males	The possibility that semaglutide reduces other types of reward was further explored in feeding experiments, where the intake of rewarding/palatable foods was measured	3	Effects on other feeding parameters at 4-h time point (Supplementary Fig. S9) and at 2-h time point (Supplementary Fig. S10).
7	Effects of alcohol/semaglutide on dopamine-related gene expression	Mice	Males	To explore if semaglutide and alcohol increases the expression of the enzymes metabolizing dopamine (MAOA, MAOB, COMT) in NAc.	4	Gene expression in the VTA (Supplementary Fig. S13).
8	Effects of semaglutide on *ex vivo* dopaminergic neurotransmission in NAcS and NAcC from alcohol drinking rats of both sexes.	Rats	Males Females	To further explore the possibility that semaglutide together with alcohol changes dopamine metabolism	Supplementary Fig. S12	Additional *ex vivo* data from other brain areas (Supplementary Fig. S13 and Supplementary Table S1–S2)
9	Effects of semaglutide on other dopamine driven behaviours, alcohol naïve	Rats	Males Females	To explore the possibility that semaglutide affects exploratory behaviours and novelty seeking.	Supplementary Fig. S12	
10	Detection of fluorescently labelled semaglutide in NACS of alcohol drinking rats	Rats	Males Females	As semaglutide increases the metabolism of dopamine in NAcS when alcohol is onboard, we hypothesise that fluorescently labelled semaglutide may be preset and exert its effects locally in NAc.	3	
11	Effects of semaglutide on cAMP in NAc, alcohol naïve	Mice	Males Females	To understand the effects of semaglutide within the NAc on cAMP, a key downstream signalling molecule of GLP-1R was measured in the NAc	Supplementary Fig. S14	cAMP in amygdala, VTA, NTS (Supplementary Fig. S14)
12	Effects of semaglutide on activity and anxiety-like behaviours	Rats	Males Females	Activity and anxiety are tentative confounding factors to the semaglutide reduced alcohol intake, a possibility explored in locomotor activity and elevated plus maze experiments	Supplementary Fig. S15	

Summary of all experiments conducted. Description of species and sex used, the rational for conducting the experiments, description figure/supplementary material where the results can be found, and additional outcome obtained (besides the main findings). Nucleus accumbens shell (NAcS) and core (NAcC), ventral tegmental area (VTA), nucleus tractus solitarius (NTS), glucagon-like peptide-1 (GLP-1R).

#### Effects of acute or repeated administration of semaglutide alcohol intake in male and female rats

As high alcohol intake over prolonged period of time is important for the manifestation of AUD, we explored the potential of semaglutide to reduce alcohol intake in male and female rats. In each alcohol drinking experiment the intermittent access model was used, where individually housed male or female rats could choose between one alcohol (20%) bottle and one water bottle for three 24-h sessions per week (Monday, Wednesday, Friday) and two water bottles the remaining days.^15^^,^^36^ The bottles were changed when lights were turned down. In this model alcohol consumption escalates to high, stable levels without using sucrose fading or forced procedures,^26^^,^^37^ and the model has been proposed to reflect several aspects of alcohol abuse.^38^ Predictive validity of this model has been suggested as acamprosate (anti-AUD medication) reduces alcohol intake in this model but not in continuous access models.^26^^,^^39^ While others report an alcohol deprivation effect in this model,^40^^,^^41^ this is not evident in our studies. In this model, we measure the intake of alcohol, water and food as well as body weight throughout the experiments, and the rat's consumed alcohol for 8–10 weeks before treatment initiation (baseline drinking). For all experiments, the baseline alcohol intake was similar between rats later assigned to semaglutide or vehicle treatment. Baseline alcohol intake was used to stratify the experimental rats, such that the vehicle-treated group was matched with the semaglutide-treated group based on similar pre-treatment drinking behaviour. The following treatment groups were used for each experiment: semaglutide or vehicle. Pre-set exclusion criterion was leaking bottles.

**Fig. 1:**
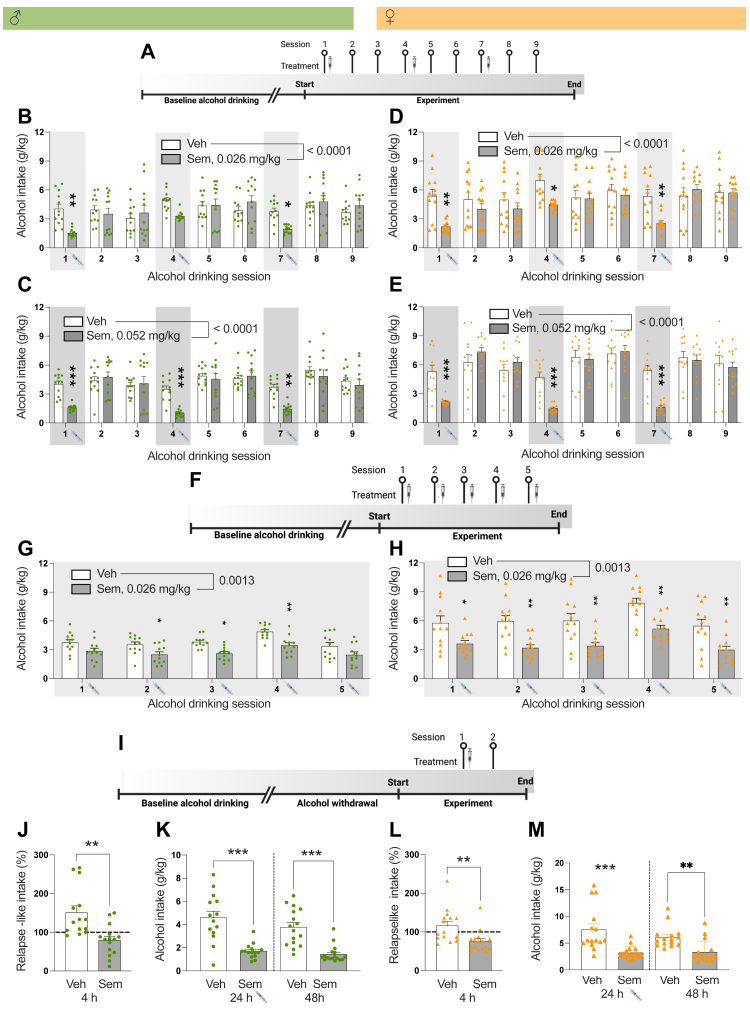
**Semaglutide treatment decreases alcohol intake and prevents relapse drinking in male and female rats.** (A) Schematic illustration of the alcohol drinking experiment, in which semaglutide was injected acutely at three alcohol drinking sessions (S1, S4, S7). (B) In male rats, semaglutide (0.026 mg/kg, n = 12) reduces the overall alcohol intake compared to vehicle (n = 12); a reduction specifically evident at alcohol drinking session 1 and 7. (C) As further shown in males, in comparison to vehicle (n = 12) semaglutide (0.052 mg/kg, n = 12) decreases alcohol intake at each treatment session. Similarly, compared with vehicle (n = 12 in each test) both semaglutide doses, (D) 0.026 mg/kg (n = 12) and (E) 0.052 mg/kg (n = 12), reduce alcohol intake at each treatment session in female rats. (F) Schematic illustration of the alcohol drinking experiment, in which semaglutide was injected repeatedly at five subsequent alcohol drinking sessions (S1, S2, S3, S4, S5). (G) In this design, repeated semaglutide (0.026 mg/kg, n = 12) treatment reduces alcohol intake in male rats compared to vehicle (n = 12). (H) Additionally, in comparison to vehicle (n = 12), repeated semaglutide treatment (0.026 mg/kg, n = 12) lowered alcohol intake in female rats. This decline is evident at drinking session 2, 3 and 4 in males, and at each session in females. (I) Schematic illustration of the alcohol drinking experiment, in which the effect of semaglutide on relapse-like drinking after alcohol withdrawal was tested. Semaglutide is injected 60 min prior to alcohol exposure, thereafter the rats consumed alcohol for 48 h. (J) The % increase in alcohol intake from baseline (dotted line) is reduced by semaglutide. Specifically, alcohol withdrawal induces a relapse drinking (t (13) = 3.29, P = 0.0059, paired t-test, n = 14) in male rats, and semagltuide blocks the relapse-like drinking (t (14) = 2.67, P = 0.0187, paired t-test, n = 15) (data not shown). (K) Additionally, semaglutide decreases the alcohol intake 24 and 48 h after treatment. (L) In female rats, semaglutide reduces the % increase in alcohol intake from baseline (dotted line). Indetail, alcohol withdrawal trends to cause relapse-like drinking (t (14) = 1.18, P = 0.1287, paired t-test, n = 14) in female rats and this was prevented by semaglutide treatment (t (15) = 4.65, P = 0.0003, paired t-test, n = 16) (data not shown). (M) The reduced alcohol intake is also evident 24 and 48 h after semaglutide treatment. Overall interaction effect (B–E) and overall treatment effect (G–H) from two-way ANOVA with repeated measures is stated in figure. J–K data were analysed with a paired t-test and K-L wih an unpared t-test. Experiments were not replicated. Data are presented as mean ± SEM, significant data are illustrated by ∗P < 0.05, ∗∗P < 0.01, ∗∗∗P < 0.001. Syringe indicates time of injection.

To investigate the acute effects of semaglutide on alcohol intake (Fig. 1A), male (n = 24) or female (n = 24) rats were treated with vehicle or semaglutide (0.026 mg/kg, sc) 60 min prior to alcohol exposure. In these initial experiments semaglutide was administered once a week (on Mondays) for three consecutive weeks in an attempt to identify the lowest effective dose for reducing alcohol intake. Subsequent experiments explored semaglutide's dose-dependent effect on alcohol drinking. Therefore, male (n = 24) or female (n = 24) rats were injected once weekly (Mondays) with a slightly higher dose of semaglutide (0.052 mg/kg) or vehicle (Fig. 1A) 60 min prior alcohol exposure.

Further studies were conducted to test the ability of repeated semaglutide to reduce alcohol intake (Fig. 1F) over a longer alcohol drinking period. Semaglutide (0.026 mg/kg) or vehicle was injected at five subsequent alcohol drinking sessions (Monday, Wednesday, Friday, Monday, Wednesday) to male (n = 24) and female rats (n = 24).

#### Effects of acute semaglutide treatment on relapse-like drinking in male and female rats

The inability of patients with AUD to abstain from alcohol during abstinence is a central phenomenon for the AUD diagnosis and involves craving for alcohol. The alcohol deprivation model can be used to reflect relapse-like drinking in male rats.^42^ The hypothesis that semaglutide prevents relapse-like drinking in the alcohol deprivation model was then tested in additional rats of both sexes (Fig. 1I). After 8 weeks of intermittent access to alcohol, male (n = 33) or female (n = 31) rats were deprived of alcohol for 9 days. 60 min prior to alcohol reintroduction, the rats were treated with vehicle or semaglutide (0.026 mg/kg, sc). Thereafter alcohol was reintroduced continually for 48 h (Monday-Tuesday). Their alcohol intake was then measured after 4 (0–4) hours, as well as 24 (0–24) and 48 (24–48) hours, after the alcohol reintroduction. Relapse-like drinking is defined as the % change from the alcohol intake during baseline. The intake as baseline and at reintroduction was also compared for both vehicle and semaglutide treated rats.

#### Effects of acute semaglutide treatment on alcohol's ability to activate the mesolimbic dopamine system in male mice

Alcohol activates the mesolimbic dopamine system causing a locomotor stimulation and dopamine release in NAcS; alcohol responses tentatively associated with reward in humans (for review see^9^). The potential of semagltuide to supress alcohol-induced locomotor stimulation and dopamine release in NAcS was therefore tested in male mice.

The 1-h locomotor activity (distance travelled) was registered in six open field boxes (42 × 42 × 20 cm; Med Associates Inc; Georgia, Vermont, USA) as described previously.^15^^,^^36^ Activity was measured by 16 × 16 infrared beams located at two different levels. Grouped-housed mice were used for the present experiment. The male mice were allocated to treatment groups using a cage-stratified approach to ensure that all treatments were represented within each cage, and a box-stratified approach to ensure that different treatment were allocated to all open filed boxes used. The following treatment groups were used: vehicle–vehicle, vehicle-alcohol, semaglutide-vehicle, semaglutide-alcohol. 12 male mice were assigned to vehicle–vehicle and semaglutide-vehicle treatment. 18 male mice were assigned to vehicle-alcohol and semaglutide-alcohol, as the response variation is higher by alcohol. In this experiment, male mice (n = 60) were injected with semaglutide (0.026 mg/kg) or vehicle. They were then allowed 60-min to habituate to the open field boxes that are ventilated, dim lit (3 lux) and sound isolated. Thereafter, the male mice were treated with alcohol (1.75 g/kg) or vehicle and the distance travelled in the open field boxes was measured. Stereotypic counts, vertical counts, and average velocity were also recorded. Pre-set exclusion criterion was intra-cage male aggressive behaviours as this negatively influences normal behaviours.

Next, the ability of semaglutide to lower the alcohol-induced dopamine release in NAcS was measured in freely moving male mice. First, by means of a surgical procedure^43^^,^^44^ a probe was inserted unilaterally in NacS (Supplementary Fig. S1) of male mice (n = 40). The mice were anesthetized using Isoflurane Baxter (administered by a Univentor 400 Anaesthesia Unit from Univentor Ldt, Zeitun, Malta). They were then placed on a heating pad in a stereotaxic frame (David Kopf Instruments, Tujunga, CA, USA). Pain relief was provided through local application of Xylocaine Adrenaline (5 μg/ml; from Pfizer Inic, New York, NY, USA) and sc injection of Caprofen (5 mg/kg; Astra Zeneca, Gothenburg, Sweden). The surgical site was prepared by exposing the skull bone and drilling a hole for the probe at coordinates relative to bregma of 1.3 mm anterior, ±0.5 mm lateral to midline, and −4.6 mm ventral, as well as one additional hole for anchoring screw. The probe was implanted unilaterally and alternated between the left and right sides of the brain. Following the surgery, the mice were held individually for two recovery days.

Thereafter the *in vivo* microdialysis was conducted^43^^,^^44^ where the following treatment groups were used; vehicle–vehicle, vehicle-alcohol, semaglutide-vehicle, semaglutide-alcohol (n = 56). Mice were allocated to treatment groups to ensure each treatment was tested in every microdialysis setup (6 at each occasion). Pre-set exclusion criteria were loss of implantation, misplaced probes and technical difficulties during microdialysis. At the test day, a microperfusion pump (U-864 Syringe Pump: AgnThós AB) was connected to the probe with the perfusion rate of 1.6 μl/min and the mice habituated for 2 h. Three baseline samples were collected and the mice were then injected with semaglutide (0.026 mg/kg) or vehicle followed by alcohol (1.75 g/kg) or vehicle. Thereafter, samples were collected every 20 min for three additional hours, allowing identification of treatment outcome over time in freely moving mice. The levels of dopamine, noradrenaline, serotonin, L-DOPA, and their metabolites (HVA, DOPAC, 3-MT, NM, 5-HIAA) were detected using a two dimensional high-performance liquid chromatography together with electrochemical detection (HPLC-EC) system, according to a modified protocol.^45^ After termination, the probe location was verified with microtome, and only mice with correct placements were included in the analysis (Supplementary Fig. S1A and B). Besides evaluting the treatment effect on the dopamine release compared to baseline, the outcome on the area under the curve (AUC) was analysed.

As memory of the alcohol reward is an important aspect of the AUD process, the effect of semaglutide on this was explored by means of the memory of CPP (mCPP) test as described previously.^15^^,^^16^^,^^30^ In brief, a two-chambered CPP apparatus with distinct tactile and visual cues was used in dim-light (3 lux). The paradigm consists of pre-conditioning (day 1), conditioning (days 2–5), and post-conditioning (day 6), where each session is 20 min. A biased design was used, in which alcohol was conditioned to the least preferred side during pre-conditioning. The injections were altered between morning an afternoon in a balanced design. After the pre-test in which the group housed male mice (n = 16) were allowed to explore the entire CPP arena after a vehicle injection, the mice were conditioned to alcohol (1.75 g/kg) for four days.^15^^,^^16^^,^^30^ At post-test the mice were injected with vehicle or semaglutide (0.026 mg/kg) and the male mice were allowed to explore the entire CPP arena. Mice were assigned to either alcohol-vehicle or alcohol-semaglutide treatments. The mice were stratified into treatment based on initial side preference and time of day (morning or afternoon) in which the alcohol conditioning occurred. Additional control mCPP tests were conducted in other male mice (n = 16), which aimed to exclude the possibility that semaglutide has an effect on mCPP *per se*. Given the long half-life of semaglutide, together with the design of CPP for alcohol, conditioned approach behaviour (*i.e,* reflection of reward) could not be tested. CPP is determined by subtracting the time spent in the alcohol-conditioned chamber during the pre-conditioning phase from that in the post-conditioning phase, and dividing the difference by the total time spent in the CPP apparatus. Jumping out of the CPP apparatus is pre-set exclusion criterion.

#### Effects of acute semaglutide treatment on the intake of rewarding/palatable foods in alcohol-naïve male mice

The possibility that semaglutide reduces other types of reward was further explored in feeding experiments, where the intake of rewarding/palatable foods^46^ was measured in alcohol-naïve male mice treated with semaglutide. As described previously,^47^^,^^48^ the group-housed mice were pre-exposed to peanut butter (Crunchy, Green Choice, 590 kcal/100 g, n = 24) or Nutella (Ferrero, 514 kcal/100 g; n = 16) in their home cage daily (4 h per day for 3 days), where they also had *ad lib* access to chow. On the experiment day, the mice were injected with semaglutide (0.026 mg/kg) or vehicle, placed in an individual novel cage where they had free access to regular food (chow) and either peanut butter or Nutella. Mice were allocated to treatment groups to ensure equal distribution the following treatment groups were used: semaglutide and vehicle. The consumption of chow and rewarding food, the preference (defined as percentual ratio of rewarding food to total food ingested), and caloric intake was measured at 2- and 4-h timepoints. Excessive bedding stuck in rewarding food is the pre-set exclusion criterion.

#### Effects of semaglutide and alcohol treatment on the expression of the enzymes metabolizing dopamine in NAc in male mice

As the *in vivo* microdialysis findings revealed that semaglutide in combination with alcohol increased the levels of the dopamine metabolites DOPAC and HVA in NAcS of male mice, we hypothesized that the treatment combination enhances the expression of the enzymes metabolizing dopamine (MAOA, MAOB, COMT) of male mice.^49^ To exclude the possibility that this effect was mediated instead via changes in reuptake, the treatment effect on expression of the dopamine reuptake transporter (DAT) was also tested. Therefore, group-housed male mice (n = 10) were injected with semaglutide (0.026 mg/kg) or vehicle followed by an alcohol (1.75 g/kg) injection. Mice were allocated to treatment groups to ensure equal distribution and the following treatment groups were used: alcohol-semaglutide and alcohol-vehicle. One hour later (corresponding to elevated DOPAC, HVA levels in the *in vivo* microdialysis experiment), the male mice were sacrificed, the brains were collected and placed in brain slicing matrix, NAcS and NAc core (NAcC) were rapidly punched out. *Ex vivo* tissue was stored in −80 °C until further qPCR analysis. Total RNA was extracted, purified and amplified as done before.^32^^,^^50^ The expression of the *MAOA*, *MAOB*, *COMT* and *DAT* genes (ThermoFisher, MAOA; Mm00558004_m1, MAOB; Mm00555412_m1, COMT; Mm00514377_m1, DAT; Mm00438396_m1; Catalogue number 4331182) was normalized to the geometric mean of beta-actin (ThermoFisher, Mm01205647_g1; Catalogue number 4331182). Thereafter, the expression *COMT, MAOA*, *MAOB* and *DAT* genes, were expressed as fold change relative to vehicle-alcohol. Besides NAc, the expression of these dopamine related genes was investigated in the area projecting to NAc, namely the ventral tegmental area (VTA). The pre-set exclusion criterion was contamination of samples.

#### Effects of semaglutide on *ex vivo* levels of dopamine metabolites in NAc in alcohol-drinking male and female rats

To further explore the potential of semaglutide together with alcohol to enhance dopamine metabolism in NAc, the *ex vivo* levels of monoamines and their metabolites were measured in NAcS and NAcC of alcohol-drinking male and female rats treated with semaglutide. While *in vivo* microdialysis assess the release of dopamine metabolites in freely moving male mice over time, *ex vivo* HPLC measures neurotransmission in one area at one time point after termination. Therefore, the *in vivo* data provides more details, and the *ex vivo* data should be considered as a complement. Thus, after repeated vehicle or semaglutide (0.026 mg/kg) treatments (5 occasions), the alcohol-drinking rats were sacrificed, brains were collected, brain areas were punched out and stored in −80 °C. The *ex vivo* levels of monoamines (dopamine, noradrenaline, serotonin), the dopamine pre-cursor (L-DOPA) and metabolites (DOPAC, HVA, 3-MT, 5-HIAA) were determined by an established HPLC-EC method.^48^ Moreover, to gain insight into neurotransmission in other reward related areas that modulate alcohol responses^35^^,^^51^, ^52^, ^53^, ^54^ the *ex vivo* levels of these neurotransmitters were analysed in VTA, laterodorsal tegmental nucleus, paraventricular thalamus and lateral septum. The pre-set exclusion criterion was contamination of samples.

#### Effects of semaglutide treatment on dopamine driven behaviours in alcohol-naïve male and female rats

To further explore the possibility that semaglutide affects dopaminergic neurotransmission, its influence on dopamine driven behaviours like exploration and novelty-seeking^24^^,^^25^ was tested in alcohol-naïve male (n = 20) and female (n = 20) rats that were group-housed. Rats were allocated to treatment groups to ensure equal distribution and the following treatment groups were used: semaglutide and vehicle. Semaglutide (0.026 mg/kg) or vehicle was injected and 60 min later the rats were placed in the centre of the open field boxes (42 × 42 × 20 cm; Med Associates Inc) that are sound isolated and dim lit (3 lux). They explored the arena for 60 min, were removed shortly and a novel object (Lego piece, 3 × 3 × 3 cm) was placed in the centre of arena. Then, the rats explored the object and arena for two additional minutes. For both tests, time spent in inner/novel zone (15 × 15 cm) and corners, as well as zone entries were analysed by Observer XT software (Noldus, Wagenegen, Netherlands). To prevent treatment bias in scoring, the person responsible for scoring was required to have an inter- and intraindividual scoring reliability of at least 90%. Pre-set exclusion criterion was intra-cage aggressive behaviours as this negatively influences normal behaviours.

#### Detection of the fluorescently labelled semaglutide in NAcS of alcohol-drinking male and female rats

As semaglutide increases the metabolism of dopamine in NAcS when alcohol is onboard, we hypothesise that fluorescently labelled semaglutide is present and possibly exerts its effects locally in this area. After 10 weeks of alcohol drinking, CY3-semaglutide or vehicle was injected acutely to male (n = 2) and female (n = 2) rats in order to investigate the possibility that CY3-semaglutide could reach NAcS. 60 min later the rats were perfused, the brains were removed and post-fixed, cut into 40 μm NAc sections and stored as described previously.^32^ The brain sections were washed with TBS (Tris-buffered saline) and mounted on adhesion glass slides (SuperFrost Plus™, ThermoFisher Scientific, Waltham, MA, USA) and coverslips using a few drops of mounting media (ProLong™ Gold Antifade, Life Technologies Europe BV). A ZEISS Axio observer Z1 with a 20× objective and the ZEN microscope software was used to image CY3-semaglutide. Blood within slices dampens the visualisation in microscopes and was therefore used as pre-set exclusion criterion.

#### Effects of semaglutide treatment on cAMP in NAc of alcohol-naïve male mice

While the *in vivo* micodialysis experiments in male mice revealed an enhanced dopamine metabolism by semaglutide together with alcohol, semaglutide did not change dopamine, DOPAC and HVA *per se*. We therefore hypothesis that semaglutide does not activate NAc neurons when interjected alone. To explore this, the effect of semaglutide (0.026 mg/kg) on cAMP, a key downstream signalling molecule of GLP-1R,^55^^,^^56^ was measured in the NAc. To explore other tentative areas targeted by semaglutide, cAMP was measured in areas projecting to NAc (amygdala, VTA or nucleus tractus solitarius (NTS)). Alcohol-naïve group-housed male mice (n = 14) were treated with vehicle or semaglutide, sacrificed after 60 min and the above-mentioned brain regions were punched out and kept in −80 °C until further ELISA (Catalog number: ADI-900-066A) analysis according to manufacturer's instructions. Mice were allocated to treatment groups to ensure equal distribution and the following treatment groups were used: semaglutide and vehicle. Contamination of samples was the pre-set exclusion criterion.

#### Effects of acute treatment with semaglutide on locomotor activity and anxiety-like behaviours in alcohol-naïve male and female rats

Activity and anxiety are tentative confounding factors to the semaglutide reduced alcohol intake, a possibility explored in locomotor activity and elevated plus maze (EPM) experiments.^57^^,^^58^ After 30-min habituation to the open field boxes (42 × 42 × 20 cm, Med Associates Inc) that are ventilated, dim lit (3 lux), alcohol-naïve male (n = 20) and female (n = 20) rats were injected with semaglutide (0.026 mg/kg) or vehicle, and their 60-min activity was recorded. Distance travelled, vertical counts, zone entries and time in inner zone were analysed. In the EPM test, alcohol-naïve male (n = 20) and female (n = 20) rats were injected with semaglutide (0.026 mg/kg) or vehicle and placed in the centre of the EPM, that consists of closed (40 cm high walls) and open arms elevated 70 cm above the floor. Time in the open and closed arms, and central zones was analysed by Observer XT software (Noldus, Wageningen, Netherlands). For both tests, the rats were allocated to treatment groups to ensure equal distribution and the following treatment groups were used: semaglutide and vehicle. Pre-set exclusion criterion was intra-cage aggressive behaviours as this negatively influences normal behaviours.

### Statistical analysis

All statistical analysis was conducted using Prism 9.0 (GraphPad Software, Inc., CA, USA). The data from the alcohol drinking experiments and the *in vivo* microdialysis experiment were analysed with a repeated two-way ANOVA, followed by the recommended post-hoc test (Tukey's or Bonferroni). The remaining experiments were analysed by unpaired t-test (two treatment groups) or one-way ANOVA (four treatment groups), followed by the recommended post-hoc test (Tukey's). The probability of P < 0.05 is considered as statistically significant.

### Role of the funding source

The funding sources did not play a role for the study design, collection, analysis, and interpretation.

## Results

### Acute and repeated semaglutide treatment decreases alcohol intake in male and female rats

The initial alcohol drinking experiments were conducted to explore the potential of semaglutide to dose-dependently reduce alcohol-drinking in rats of both sexes.

Both semaglutide doses decreased alcohol intake in male (Fig. 1B and C) and female (Fig. 1D and E) rats. In male rats, compared to vehicle (n = 12) there was an overall interaction (F(8,176) = 7.64, P < 0.0001) and time (F(8,176) = 8.44, P < 0.0001), but not treatment (F(1,22) = 0.69, P = 0.4160), effect on alcohol intake by semaglutide (0.026 mg/kg, n = 12) (two-way ANOVA, with repeated measures). Specifically, this decrease was evident at alcohol drinking session 1 and 7 (P < 0.05, S7; P < 0.001, S1; Tukey's posthoc test). Additionally in males, in comparison to vehicle (n = 12) there was an overall interaction (F(8,176) = 7.81, P < 0.0001), time (F(8,176) = 24.43, P < 0.0001) and tend toward treatment (F(1,22) = 3.86, P = 0.0623) effect on alcohol drinking by semaglutide (0.052 mg/kg, n = 12) (two-way ANOVA, with repeated measures). The lowered alcohol intake was shown at each alcohol session (P < 0.001, S7; P < 0.0001, S1, S4; Tukey's posthoc test). Moreover, semaglutide (0.026 mg/kg) decreased the preference for alcohol, without altering the water intake or total fluid intake and reduced body weight (Supplementary Fig. S2A–D, I). It is further found in males that the higher semaglutide dose (0.052 mg/kg) had an overall interaction effect on alcohol preference, and that the posthoc tested revealed a lowered alcohol preference at session 8 and trend towards a reduction at session 5 (Supplementary Fig. S2E and I). Moreover, semaglutide (0.052 mg/kg) decreased water, total fluid and the body weight in males (Supplementary Fig. S2F–H and I).

As in male rats, both semaglutide doses reduced alcohol intake in female rats (Fig. 1D–E). Specifically, there was an overall interaction (F(8,176) = 7.86, P < 0.0001) and time (F(8,176) = 10.71, P < 0.0001), but not treatment (F(1,22) = 2.64, P = 0.1183), effect on alcohol consumption by semaglutide (0.026 mg/kg, n = 12) compared to vehicle (n = 12) (two-way ANOVA, with repeated measures). This reduction was observed at each alcohol drinking session (P < 0.05, S4; P < 0.001, S1, S7; Tukey's posthoc test). Moreover, semaglutide (0.052 mg/kg, n = 12) had an overall interaction (F(8,176) = 14.30, P < 0.0001) and time (F(8,176) = 41.22, P < 0.0001), but not treatment (F(1,22) = 2.39, P = 0.1363), effect on alcohol intake compared to vehicle (n = 12) (two-way ANOVA, with repeated measures). The ability of semaglutide to reduce alcohol intake was found at each alcohol drinking session (P < 0.0001, S1, S4, S7; Tukey's posthoc test). Both doses of semaglutide decreased the preference for alcohol, increased both water intake, and total fluid intake as well as lowered the overall body weight in female rats (Supplementary Fig. S3A–H and I).

For both male and female rats, alcohol intake did not differ between treatment groups on days without semaglutide injections, suggesting that the drug was fully metabolised between sessions (data not shown).

In rats of both sexes, the dose of 0.052 mg/kg reduced alcohol consumption more than 0.026 mg/kg (Supplementary Fig. S4A, B and M). This dose-response effect was also true for food intake, but not for the body weight reduction (Supplementary Fig. S4C–F and M). Moreover, the dose of 0.026 mg/kg reduced alcohol intake and food intake with the same magnitude in both sexes (Supplementary Fig. S4G, H and M). For this dose there was an overall interaction effect on body weight between sexes, where the reduction tended to be higher in males (Supplementary Fig. S4I and M). The dose of 0.052 mg/kg decreased alcohol intake more in females, while it decreased food intake and body weight to a greater extent in males (Supplementary Fig. S4J–L and M).

Repeated semaglutide treatment reduced alcohol intake in both sexes (Fig. 1G–H). In male rats, there was an overall treatment (F(1,22) = 13.46, P = 0.0013) and time (F(4,88) = 12.45, P < 0.0001), but not interaction (F(4,88) = 0.63, P = 0.6421), effect on alcohol drinking by semaglutide (n = 12) compared to vehicle (n = 12) (two-way ANOVA with repeated measures). This decline was evident at drinking session 2, 3 and 4 (P < 0.05, S2, S3; P < 0.01, S4; Tukey's posthoc test). Moreover, in males, repeated semaglutide treatment neither altered the alcohol preference or water intake, but had an overall reduction on total fluid intake and decreased the body weight (Supplementary Fig. S5A–D and I).

In female rats, semaglutide (n = 12) had an overall treatment (F(1,22) = 13.68, P = 0.0013) and time (F(4,88) = 26.61, P < 0.0001), but not interaction (F(4,88) = 0.47, P = 0.7553), effect on alcohol drinking compared to vehicle (n = 12) (two-way ANOVA with repeated measures). Semaglutide lowered alcohol consumption at each session in females (P < 0.05, S1; P < 0.01, S2, S3, S4, S5; Tukey's posthoc test). Additionally, in females, repeated semaglutide treatment decreased alcohol preference, and body weight, and increased water intake and tended to enhance total fluid intake (Supplementary Fig. S5E–H and I).

Just as for acute treatment, the ability of repeated semaglutide to reduce alcohol drinking or food intake, was similar between sexes, whereas the body weight reduction was more evident in males (Supplementary Fig. S6A–C and D). No animals were excluded in these initial alcohol drinking experiments. While each experiment was only conducted once, the use of different doses and acute versus repeated treatment show the same treatment outcome.

### Semaglutide treatment prevents alcohol drinking after withdrawal in alcohol-drinking male and female rats

An essential characteristic of AUD patients is their inability to refrain from alcohol consumption during abstinence, making it a critical feature to address in treatment. Therefore, the ability of semaglutide to prevent relapse-like drinking in the alcohol deprivation model was tested in alcohol exposed rats of both sexes. In males, withdrawal induced a relapse drinking (t (13) = 3.29, P = 0.0059, n = 14; paired t-test), and this relapse-like drinking was blocked by semaglutide (t (14) = 2.67, P = 0.0187, n = 15; paired t-test) (data not shown). Specifically, the % increase in alcohol intake from baseline was reduced by semaglutide (t (27) = 3.66, P = 0.0011; Fig. 1J; unpaired t-test). Moreover, the alcohol intake was lower in male rats treated with semaglutide at 24 (t (27) = 5.09, P < 0.0001, unpaired t-test) and 48 (t (27) = 5.03, P < 0.0001, unpaired t-test) hours after treatment (Fig. 1K). In this alcohol deprivation experiment, semaglutide decreased alcohol preference and food intake, increased water and total fluid intake, whereas it reduced body weight change in males (Supplementary Fig. S7A–M and AB). Two vehicle and two semaglutide treated male rats were excluded due to leaking bottles.

In female rats, alcohol withdrawal showed a trend towards relapse-like drinking (t (14) = 1.18, P = 0.1287, n = 14, paired t-test) and this was not evident after semaglutide treatment (t (15) = 4.65, P = 0.0003, n = 16; paired t-test) (data not shown). In addition, the % increase in alcohol intake from baseline was reduced by semaglutide (t (29) = 3.14, P = 0.0039, unpaired t-test; Fig. 1L). Similarly, semaglutide lowered the alcohol intake 24 (t (29) = 3.99, P = 0.0004) and 48 (t (29) = 3.79, P = 0.0007) hours after treatment (Fig. 1M; unpaired t-test). Furthermore, in females semaglutide decreased alcohol preference and food intake, increased water and total intake, whereas it lowered the body weight change (Supplementary Fig. S7N–Z and AB). One vehicle treated female rat was excluded due to leaking bottles.

### Semaglutide decreases food intake in both male and female alcohol-drinking rats

**Fig. 2:**
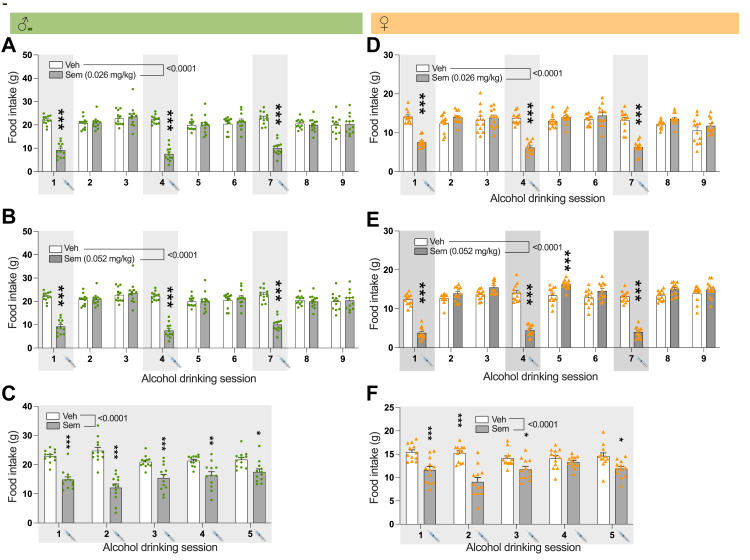
**Semaglutide reduces the intake of food in alcohol drinking male and female rats.** In male rats, semaglutide at both doses, (A) 0.026 mg/kg (n = 12) and (B) 0.052 mg/kg (n = 12) reduce the overall food intake compared to vehicle (n = 12 in each test); a reduction evident at each treatment session. (C) Compared to vehicle (n = 12), repeated semaglutide treatment (0.026 mg/kg) lowers the overall intake of food in male rats. This decline is found at each treatment session. Similarly, compared with vehicle (n = 12 in each test) both semaglutide doses, (D) 0.026 mg/kg (n = 12) and (E) 0.052 mg/kg (n = 12), decrease food intake at each treatment drinking session in female rats. (F) Additionally, in comparison to vehicle (n = 12), repeated semaglutide treatment (0.026 mg/kg, n = 12) decreases the food intake in females, a reduction evident at alcohol drinking session 1, 2, 3 and 5. Overall interaction effect (A–F) from two-way ANOVA with repeated measures is stated in the figure. Experiments were not replicated Data are presented as mean ± SEM, significant data are illustrated by ∗P < 0.05, ∗∗P < 0.01, ∗∗∗P < 0.001. Syringe indicates time of injection.

Acute or repeated treatment with semaglutide, at both at doses, decreased food intake in male (Fig. 2A–C) and female (Fig. 2D–F) rats. In males, there was an overall effect on food intake by 0.026 mg/kg of semaglutide (time, F(8,176) = 33.75, P < 0.0001; treatment, F(1,22) = 24.45, P < 0.0001; interaction, F(8,176) = 52.38, P < 0.0001; two-way ANOVA, with repeated measures). Specifically, this decrease was evident at all alcohol drinking sessions (P < 0.001; Tukey's posthoc test). It was further evident in males, that there was an overall effect on food intake by 0.052 mg/kg of semaglutide (time, F(8,176) = 85.12, P < 0.0001; treatment, F(1,22) = 50.40, P < 0.0001; interaction, F(8,176) = 84.29, P < 0.0001; two-way ANOVA, with repeated measures). This reduction was evident at all alcohol drinking sessions (P < 0.001; Tukey's posthoc test). Additionally in males, repeated treatment with semaglutide (0.026 mg/kg) had an overall treatment (F(1,22) = 41.03, P < 0.0001), interaction (F(4,88) = 11.59, P < 0.0001), but not time (F(4,88) = 1.15, P = 0.3391), effect on food intake (two-way ANOVA, with repeated measures). The lowered food intake was found at each treatment session (P < 0.05, S5; P < 0.01, S4; P < 0.0001, S1, S2, S3 Tukey's posthoc test).

In female rats, there was an overall effect on food intake by 0.026 mg/kg of semaglutide (time, F(8,176) = 19.30, P < 0.0001; treatment, F(1,22) = 13.70, P = 0.0012; interaction, F(8,176) = 29.28, P < 0.0001; two-way ANOVA, with repeated measures). Specifically, this decrease was evident at all alcohol drinking sessions (P < 0.001; Tukey's posthoc test). An overall effect on food intake by 0.052 mg/kg of semaglutide was further found in females (time, F(8,176) = 77.28, P < 0.0001; treatment, F(1,22) = 29.02, P < 0.0001; interaction, F(8,176) = 71.93, P < 0.0001; two-way ANOVA, with repeated measures). This reduction was evident at all alcohol drinking sessions (P < 0.001; Tukey's posthoc test). On a similar note, repeated treatment of semaglutide (0.026 mg/kg) decreased the intake of food (time, F(4,88) = 2.82, P = 0.0296; treatment, F(1,22) = 26.49, P < 0.0001; interaction, F(4,88) = 7.64, P < 0.0001). The lowered food intake was evident at each alcohol drinking session (P < 0.05, S3, S5; P < 0.0001, S1, S2; Tukey's posthoc test).

### Semaglutide reduces alcohol-induced activation of the mesolimbic dopamine system in male mice

The hypothesis that semaglutide inhibits alcohol-induced hyperlocomotion and lowers the dopamine levels in NAcS was tested in male mice. This was based on previous findings that alcohol consumption increases dopamine levels in the NAcS in both humans and rodents, which is linked to locomotor stimulation in mice.^4^, ^5^, ^6^, ^7^, ^8^ Moreover, this dopamine increase is associated with alcohol's rewarding experience in humans and contributes to an increased risk for an AUD diagnosis later on.^3^^,^^10^ Intriguingly, semaglutide blocked the alcohol-induced locomotor stimulation and dopamine release in NAcS in male mice (Fig. 2A–B).

Supportively, there was an overall effect (F(3,53) = 8.20, P = 0.0001; one-way ANOVA) on locomotor activity after acute administration of semaglutide followed by alcohol treatment. Compared to vehicle (n = 12), alcohol (n = 18) caused a locomotor stimulation (P = 0.0015; Bonferroni posthoc test). This locomotor stimulation was blocked by semaglutide (P = 0.0027; Bonferroni posthoc test, n = 15). Semaglutide (n = 12) had no effect on locomotor activity compared to vehicle (P > 0.999; Bonferroni posthoc test). Although this locomotor activity test was not replicated, internal-replication should be considered. Indeed, each run tests 6 mice, allowing for 10 replicates with similar treatment response between each run. Three male mice were excluded from the semaglutide-alcohol group due to pre-set exclusion criteria. The male mice had similar level of activity at habituation (Supplementary Fig. S8A and E). Effects of alcohol and semaglutide on other locomotor activity parameters are shown in Supplementary Fig. S8B–D and E.

In male mice, acute administration of semaglutide followed by an alcohol injection had an overall effect on the *in vivo* levels of dopamine in NAcS (time, F(14,504) = 2.57, P = 0.0014; treatment, F(3,36) = 7.91, P = 0.0004; interaction, F(42,504) = 2.03, P = 0.0002; two-way ANOVA with repeated measures). Compared to vehicle (n = 12), alcohol (n = 11) caused a dopamine release (40 min, P = 0.0003; 60 min, P < 0.0001; 80 min, P = 0.0009; 100 min, P < 0.0001; 120 min, P = 0.0006; 140 min, P = 0.0133; 180 min, P < 0.0001; Tukey's posthoc test). Semaglutide (n = 8) lowered the dopamine release in NAcS after alcohol (60 min, P = 0.0401; 180 min, P = 0.0327; Tukey's posthoc test). Moreover, semaglutide did not affect activity or dopamine *per se* (n = 9, Tukey's posthoc test). Moreover, there was an overall effect on AUC (F(3,36) = 6.36, P = 0.0014, two-way ANOVA; data not shown), where alcohol increased the AUC of the dopamine release (P = 0.0021, Bonferroni posthoc test), and semaglutide did not alter dopamine *per se* (P > 0.999, Bonferroni posthoc test) and tended to reduce the alcohol-induced increase (P = 0.1681, Bonferroni posthoc test). 14 mice were excluded due to pre-set criteria. While these experiments were not replicated, intra-experiment replication should be considered as each experiment tests 6 mice and there was a similar treatment response between each run.

As further shown in male mice, semaglutide (10.6 ± 5.0%, n = 8) did not inhibit the memory consolidation of alcohol reward (9.5 ± 4.1%, n = 8) in the CPP paradigm (t_14_ = 1.70, P = 0.8677), at a dose of semaglutide that did not affect the mCPP *per se* (t_14_ = 0.32, P = 0.7547; vehicle, 0.4 ± 4.5%; semaglutide −1.7 ± 4.7%, n = 8 per group). No animals were excluded due to pre-set exclusion criterion and these experiments were not replicated.

### Semaglutide decreases the consumption of rewarding foods in alcohol-naïve male mice

**Fig. 3:**
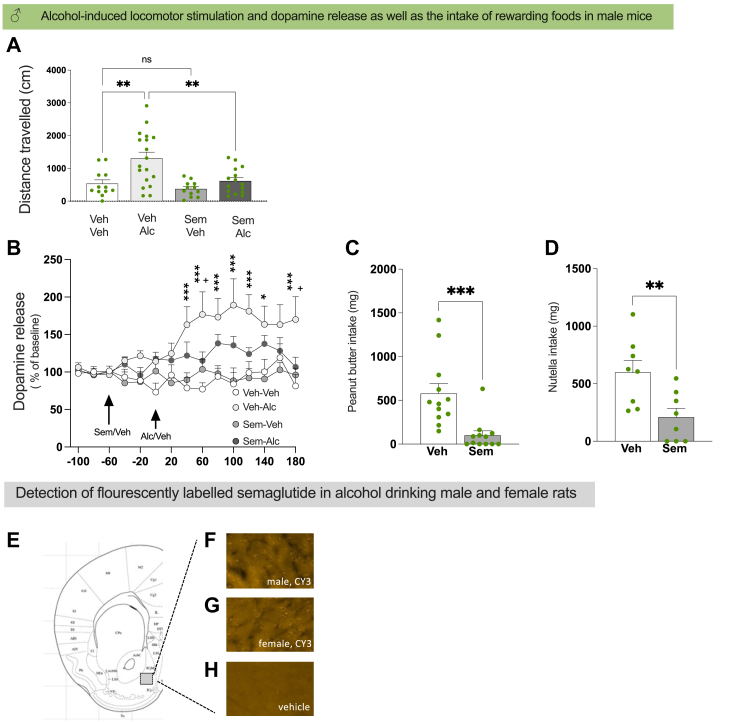
I**n male mice, semaglutide attenuates the alcohol-induced locomotor stimulation and dopamine release in nucleus accumbens shell as well as decreases the consumption of rewarding foods.** (A) Compared to vehicle (n = 12), alcohol (n = 18) causes a locomotor stimulation, and acute administration of semaglutide (0.026 mg/kg, n = 15) blocks the alcohol-induced locomotor stimulation in male mice. Additionally, semaglutide (n = 12) does not affect the activity of mice *per se*. (data analysed with one-way ANOVA) (B) In comparison to vehicle (n = 12), alcohol (n = 11) enhances the dopamine levels in nucleus accumbens shell of male mice. Semaglutide (n = 8) attenuates the ability of alcohol to increase dopamine in male mice, while it (n = 9) alone does not alter the dopamine levels. Data analysed with a two-way ANOVA with repeated measures. Together these findings indicate that semaglutide supresses the reward-like behaviours associated with alcohol of male mice. In further support for semaglutide's ability to suppress reward, it reduces the 4-h intake of (C) peanut butter (n = 12 per group) and (D) Nutella (n = 8 per group) in male mice. Data analysed with unpaired t-test. These findings have led to the hypothesis that semaglutide acts within the nucleus accumbens shell. (E) Illustration of the selected part of nucleus accumbens shell where the fluorescently labeled semaglutide (CY3-semaglutide) is detected. After systemic administration, CY3-semaglutide is detected in nucleus accumbens shell of alcohol drinking (F) male and (G) female rats. (H) This signal not found in alcohol drinking rats injected with vehicle. Experiments were not replicated. Data are presented as mean ± SEM, significant data are illustrated by ∗P < 0.05, ∗∗P < 0.01, ∗∗∗P < 0.001. +P < 0.05, ++P < 0.001 when comparing vehicle-alcohol versus semaglutide-alcohol in the microdialysis experiment.

In an attempt to further support semaglutide's ability to suppress reward-associated behaviours, experiments were conducted on alcohol-naïve male mice to observe their responses to rewarding food^46^ in the presence of semaglutide. Supportively, semaglutide lowered the 4-h intake of peanut butter (t (22) = 3.85, P = 0.0009, unpaired t-test; n = 12 per treatment group) and Nutella (t (14) = 3.03, P = 0.0090, unpaired t-test; n = 8 per treatment group) (Fig. 3C–D). Although semaglutide in both experiments increased chow intake as a compensation, it reduced the total caloric intake and the preference for rewarding foods (Supplementary Fig. S9A–G). Furthermore, semaglutide had similar effects on the 2-h timepoint (Supplementary Fig. S10A–I). No animals were excluded due to the pre-set exclusion criterion and these experiments were not replicated.

### Fluorescently labelled semaglutide is detected in NAcS in alcohol-drinking male and female rats

As semaglutide attenuates dopamine release in NAcS by alcohol, the possibility that CY3-semaglutide reaches NAcS after its systemic administration was imaged in alcohol-drinking male and female rats. In this experiment, a CY3-semaglutide signal was detected in NAcS while no CY3-semaglutide signal was detected after vehicle injection (Fig. 3E–H).

### Semaglutide enhances metabolism of dopamine in NAc of male mice when alcohol is onboard

As semaglutide supressed the ability of alcohol to activate the mesolimbic dopamine system in male mice and as CY3-semaglutide was detected in NAcS, the possibility that semaglutide augments the dopamine metabolism in NAc was tested.

**Fig. 4:**
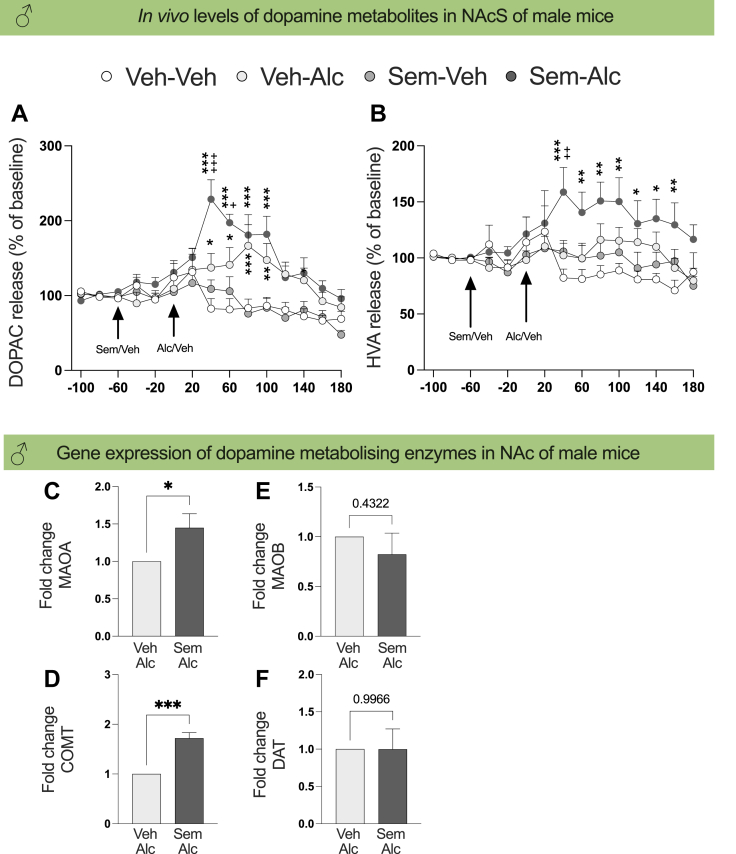
**Semaglutide enhances dopamine metabolism in nucleus accumbens when alcohol is onboard.** The *in vivo* microdialysis experiment reveals that the (A) DOPAC and (B) HVA levels in nucleus accumbens (NAc) shell are higher in male mice treated with the combination of semaglutide and alcohol compared to those treated with only alcohol (n = 12 for vehicle–vehicle, n = 18 for vehicle-alcohol, n = 12 for semaglutide-vehicle, n = 15 for semaglutide-alcohol; two-way ANOVA with repeated measures). These results indicate that semaglutide enhances the metabolism of dopamine when alcohol is onboard. In support, the NAc gene expression of (C) *MAOA* and (D) *COMT*, but not (E) *MAOB* and (F) *DAT*, is higher in male mice treated with both semaglutide and alcohol compared to those treated with alcohol (n = 5 for both treatment groups, unpaired t-test). Experiments were not replicated. Data are presented as mean ± SEM, significant data are illustrated by ∗P < 0.05, ∗∗P < 0.01, ∗∗∗P < 0.001 in comparison to vehicle treatment; +P < 0.05, ++P < 0.01, +++P < 0.001 when comparing alcohol treatment to the combination of semaglutide and alcohol.

By means of *in vivo* microdialysis experiments (number of animals identical to above *in vivo* experiments, and no replication of experiment), the levels of dopamine metabolites were measured in NAc of semaglutide treated male mice with alcohol onboard. In male mice treated with semaglutide together with alcohol, there was an overall effect on DOPAC levels in NAcS (treatment, F(3,36) = 6.10, P = 0.0018; time, F(14,504) = 10.17, P < 0.0001; interaction, F(42,504) = 3.92, P < 0.0001, two-way ANOVA with repeated measures; Fig. 4A). Alcohol increased DOPAC levels compared to vehicle (40 min, P = 0.0247, 60 min, P = 0.0120; 80 min, P = 0.0001; 100 min, P = 0.0089; Tukey's posthoc test). An increase was also evident in male mice treated with both semaglutide and alcohol (40 min, P < 0.0001; 60 min, P < 0.0001; 80 min, P < 0.0001; 100 min, P < 0.0001; 140 min, P = 0.0247; Tukey's posthoc test). Compared to alcohol alone, the combination of semaglutide and alcohol elevated DOPAC (40 min, P < 0.0001; 60 min, P = 0.0308; Tukey's posthoc test). Similarly, there was an overall effect on HVA levels in NAcS of male mice treated with semaglutide together with alcohol (treatment, F(3,36) = 10.47, P = 0.0468; time, F(14,504) = 2.85, P = 0.0004; interaction, F(42,504) = 1.92, P = 0.0007; two-way ANOVA with repeated measures; Fig. 4B) in male mice. Compared to vehicle (40 min, P < 0.0001; 60 min, P = 0.0040; 80 min, P = 0.0011; 100 min, P = 0.0026; 120 min, P = 0.0233; 140 min, P = 0.0107; 160 min, P = 0.0048; Tukey's posthoc test) or alcohol (40: P = 0.0071; Tukey's posthoc test), the combination of semaglutide and alcohol enhanced the HVA levels in NAcS. Semaglutide alone did neither affect DOPAC or HVA.

In separate experiment of male mice, the expression of enzymes degrading dopamine was measured as an increased expression of dopamine metabolising enzymes could explained the increase in dopamine metabolites. In these experiments, semaglutide together with alcohol increased the NAc gene expression of *MAOA* (t (8) = 2.38, P = 0.0447, unpaired t-test) and *COMT* (t (8) = 6.41, P = 0.0002, unpaired t-test), but not *MAOB* (t (8) = 0.83, P = 0.4322, unpaired t-test) or *DAT* (t (8) = 0.0002, P = 0.9966, unpaired t-test) compared to alcohol (Fig. 4C–F, n = 5 for each treatment group). No animals were excluded due to pre-set exclusion criterion and experiments were not replicated.

### Semaglutide influences the metabolism of dopamine in NAc of alcohol-drinking male and female rats and enhances dopamine driven behaviours in alcohol naïve male and female rats

To confirm the enhanced dopamine metabolism observed in male mice, *ex vivo* levels of dopamine and its metabolites were measured in NAc in alcohol-drinking male and female rats treated with semaglutide. Semaglutide enhanced *ex vivo* dopaminergic neurotransmission in NAcS and NAcC (Panel A, Supplementary Fig. S12A–X) of alcohol-drinking rats of both sexes. Effects of semaglutide on dopamine driven behaviours^24^^,^^25^ was tested to support the ability of semaglutide to influence dopamine signalling. Supportively, in alcohol-naïve rats of both sexes semaglutide increased exploration and novelty-seeking (Panel B, Supplementary Fig. S12A–L). No animals were excluded due to pre-set exclusion criterion (n = 10 per treatment group) and the experiments were not replicated.

### Effects of semaglutide and alcohol on neurotransmission and gene expression in other brain regions

Further data was obtained from the above experiments and these were analysed to explore the possibility of semaglutide to alter neurotransmission in other brain areas (n = 5–7, samples excluded due to pre-set exclusion criterion). Supplementary material summarises the effects of i) effects on monoaminergic neurotransmission in NAcS and NAcC of alcohol-drinking male and female rats (Supplementary Table 1), ii) semaglutide and alcohol treatment on gene expression in the VTA of male mice (Supplementary Fig. S13A–D and Q), iii) semaglutide treatment on *ex vivo* neurotransmission in VTA of alcohol-drinking rats of both sexes (Supplementary Fig. S13E–P and Q and Supplementary Table S1) and iv) monoaminergic neurotransmission in laterodorsal tegmental area, paraventricular thalamus, lateral septum from alcohol-drinking male and female rats (Supplementary Table S2). From these additional analysis VTA of male mice/rats was found as one region of interest. Experiments were not replicated.

The *in vivo* microdialysis experiment indicated that male mice's neurotransmission in NAc remained unaffected by semaglutide administration alone. We therefore hypothesised that semaglutide does not activate neurons in NAc when given alone. Supportively, semaglutide did not affect cAMP in NAc of male mice, while it tended to increase cAMP in amygdala, without affecting cAMP in VTA or NTS (Supplementary Fig. S14A–D and E, 6 and 7 per treatment group). Three samples excluded due to the pre-set exclusion criterion. Experiments were not replicated.

### Semaglutide influences anxiety-like behaviours in alcohol naïve female rats

Semaglutide effects on activity and anxiety-like behaviours were tested as such behaviours are tentative confounding factors towards semaglutide's ability to reduce alcohol intake. In alcohol naïve rats with a similar activity during habituation, semaglutide increased distance travelled, vertical counts and average velocity in males and elevated time in inner zone in females in a locomotor activity test (Supplementary Fig. S15A–L and S; n = 10 per treatment group). Although semaglutide did not influence anxiety-like behaviours of male rats, it decreased time in centre and increased time in closed arms in females in an EPM test (Supplementary Fig. S15M–R and S; n = 10 per treatment group). No animals were excluded due to pre-set exclusion criterion, and experiments were not replicated.

## Discussion

In this study, we present a diverse range of behavioural, molecular, and neurochemical evidence that supports the ability of semaglutide to significantly reduce alcohol-related responses in male and female rats, highlighting its potential as a promising treatment option for AUD.

While previous research demonstrated that a single injection of a higher dose of semaglutide (0.1 mg/kg) can decrease alcohol consumption in male rats,^23^ our study builds upon these findings. Specifically, we provide new evidence that acute treatment with low semaglutide doses (0.026 and 0.052 mg/kg) profoundly and dose-dependently suppressed alcohol drinking in both male and female rats. Besides establishing a dose-response effect, these data defined a low dose that reduced alcohol drinking that potentially may be associated with less side-effects. Similarly, the decline in alcohol intake in both sexes was found throughout the repeated semaglutide treatment period. Although a profound reduction over time shows promise for the treatment of patients with AUD, further studies are necessary to evaluate the efficacy of semaglutide when administered for prolonged periods. Given that high intake over prolonged period of time is important for the manifestation of the disease, we hypothesise that semaglutide might be helpful for treatment of AUD. While a profound and similar decrease in alcohol intake was observed by the 0.026 mg/kg dose in rats of both sexes, the reduction was more evident in females after the higher dose (0.052 mg/kg). A sex-dependent effect was also evident by dulaglutide, another GLP-1R agonist, as its alcohol lowering ability is more pronounced in males compared to females.^17^ Moreover, semaglutide reduced the preference for alcohol in both sexes. Specifically, semaglutide at 0.026 mg/kg dose acutely decreased alcohol preference in male and female rats. Repeated treatment with this dose tended to decrease preference in males and significantly reduced it in females. The 0.052 mg/kg dose lowered preference in females, but the outcome was unclear in males as it declined both alcohol and water intake. Alcohol withdrawal induced relapse-like drinking in male rats and tended to increase it in females, and semaglutide prevented this relapse-like drinking in both males and females. This finding might be relevant in humans as the inability to abstain from alcohol is a central aspect for patients with AUD. As further found in this relapse-like drinking experiment, the ability of semaglutide to reduce alcohol intake was evident both at 24 and 48 h after treatment. These findings are consistent with previous studies with other GLP-1R agonists in rodents^15^, ^16^, ^17^^,^^59^ and in overweight AUD patients,^19^ and they may therefore be indicative of a beneficial clinical use. Furthermore, semaglutide can be administered orally once weekly, an advantage for the prospective use in patients with AUD.^60^ One additional benefit of semaglutide is that, similar to dulaglutide, liraglutide, and exendin-4,^15^^,^^17^^,^^28^ it did not exhibit tolerance towards its alcohol suppressing properties. On the other hand, five treatment sessions might be too few to identify tolerance, however this was not the case for liraglutide.^15^ It should also be noted that rats, rather than mice were used for the present drinking experiments. Although up-coming experiments should evaluate the impact of semaglutide on alcohol drinking in mice, a similar outcome is expected as another GLP-1R agonist, exendin-4, reduces alcohol intake in both species.^16^^,^^18^^,^^27^, ^28^, ^29^^,^^61^ On a similar note, pharmaceuticals targeting ghrelin, another the gut-brain, display a similar outcome in rats and mice.^30^^,^^62^, ^63^, ^64^

Here, we demonstrated in male mice that semaglutide attenuated the alcohol-induced locomotor stimulation and lowered the release of dopamine in NacS after alcohol, a finding in accordance with data on other GLP-1R agonists.^15^^,^^16^^,^^65^ We therefore suggest that semaglutide's ability to suppress alcohol-induced activation of the mesolimbic system, and tentatively reward, is one aspect that contribute towards its ability to reduce alcohol consumption and prevent relapse-like drinking. As reward cannot be directly measured in rodents, human studies are warranted to investigate the potential of semaglutide on the rewarding experience of alcohol. Notably, these findings might be relevant as the experience of alcohol reward is a risk factor for AUD diagnosis later in life.^10^ It should however be noted that reward is one aspect controlling alcohol consumption and factors like impulsivity, stress reactivity and continued intake despite negative consequences also modulate alcohol drinking. Up-coming rodent and human tests should therefore explore the potential of semaglutide to modulate such factors. The ability of semaglutide to suppress activation of the mesolimbic dopamine system, and tentatively reward was further evident as it reduced both the intake and preference for rewarding foods in alcohol-naïve male mice. On a similar note, exendin-4 reduces the consumption of rewarding foods and the reward thereof.^61^^,^^66^, ^67^, ^68^, ^69^, ^70^, ^71^ It should however be noted that semaglutide also increased the chow intake, an outcome plausibly due to compensation for the decrease in rewarding foods. Since semaglutide decreases chow intake in situations where no rewarding food is available,^35^^,^^72^ the present data implies that semaglutide reduces the intake of the most rewarding food in a choice situation. The exclusion of female mice in these mice experiments is a limitation, as the possibility of sex-specific effects of semaglutide on these behaviours cannot be ruled out.

While we suggest that semaglutide reduces alcohol consumption due to reward attenuation, at least in males, other factors might influence the obtained data. One of these is malaise, a common side effect by GLP-1R agonists,^73^ including semaglutide.^74^ However, this appears less likely as semaglutide blocked the alcohol-induced hyperlocomotion and lowered the elevated dopamine levels in NacS, which both are alcohol-related responses not affected by malaise.^4^^,^^6^^,^^75^, ^76^, ^77^ As low doses of semaglutide do not cause malaise in one, but not in another study,^74^^,^^78^ the effects of low semaglutide doses on conditioned taste aversion or pica intake in rodents exposed to alcohol should be explored. Another tentative confounding factor is memory alteration, which is improbable as we show that semaglutide did not alter the memory of alcohol reward in the CPP test. While sedation could potentially account for the reduced alcohol consumption observed, it is improbable due to the increased activity observed during locomotor activity testing with semaglutide administration alone. Although not investigated in this study, increased stress is unlikely to be a contributing factor as all GLP-1R agonists tested decrease alcohol consumption, but either increase or decrease corticosterone.^79^^,^^80^ As opposed to exendin-4 and GLP-1,^81^ semaglutide slightly enhanced anxiety-like behaviours in female rats which might influence the obtained data and further suggests that anxiety levels should carefully be monitored in anxiety prone patients, and in particular women, treated with semaglutide. The possibility that the caloric content of alcohol influences the suppressed alcohol consumption by GLP-1R agonists is less likely as they show similar effects on drugs that, of course, do not have calories.^65^, ^66^, ^67^, ^68^, ^69^ Although the semaglutide solution contained other substances that could potentially affect the obtained data, the extensive dilution makes it less likely to have had a significant impact.

The present data further demonstrated that semaglutide together with alcohol enhanced the dopamine metabolism in NAc of male subjects. In male mice, the combined treatment of alcohol and semaglutide resulted in an increase in the *in vivo* levels of both DOPAC and HVA in NAcS, which may be attributed to the upregulated gene expression of *MAOA* and *COMT* in NAc. Although tested at different conditions, similarities between rats and mice in this regard appear to exist. Indeed, alcohol-drinking male rats treated with semaglutide displayed enhanced *ex vivo* levels of dopamine metabolites in both NAcS and NAcC. The elevated dopamine metabolism evident in male mice and rats may be due to a direct effect of semaglutide in NAc. Supportively, fluorescently labelled semaglutide was detected in this region in alcohol-drinking male rats after its systemic administration. It should however be noted that semaglutide did not increase cAMP, dopamine, HVA or DOPAC in NAc of alcohol-naïve male mice, indicating that alcohol is necessary for semalgutide to alter dopamine metabolism. Given that dopamine in NAc is associated to alcohols rewarding properties^3^ and that treatment-induced enhancement of dopamine metabolism reduces dopamine levels in NAc, we postulate that semaglutide attenuates the reward-like responses to alcohol. The NAc receives dopaminergic innervation from the VTA, a region mediating reward-related processes and the dopamine release in NAc.^59^^,^^69^^,^^82^ It is therefore of interest that the combination of alcohol and semaglutide reduced genes expression of dopamine metabolising enzymes in male mice and decreased the *ex vivo* levels in alcohol-drinking male rats. A reduced dopamine tone within the VTA may thus also contribute to semaglutide's ability to suppress alcohol-related responses. The findings that semaglutide increased dopamine-driven behaviours such as exploration and novelty-seeking^83^ provide further support for its interaction with dopamine signalling.

It should be further noted that semaglutide attenuated the alcohol-induced dopamine release through enhanced dopamine metabolism rather than increased dopamine reuptake. Notably, the gene expression of *DAT* was unaffected by treatment in male mice. The effects of semaglutide and alcohol on *in vivo* levels of dopamine metabolites and gene expression were not studied in female rats, and different mechanisms may be relevant for females. However, it is worth noting that the potential relevance of the interaction between semaglutide, alcohol and dopamine for females cannot be dismissed. Supportively, *ex vivo* neurochemical data demonstrated changes in dopamine metabolism in the NAc of female rats that consumed alcohol. Additionally, after systemic administration of fluorescently labelled semaglutide, it was detected in NAc of female rats exposed to alcohol.

In the present study, the fluorescently labelled semaglutide was detected in NAcS of both male and female alcohol-drinking rat, indicating that semaglutide exerts its effects locally in the NAc. As it previously has been suggested that semaglutide mainly penetrates circumventricular organs and that its distribution is limited,^35^ we suggest an increased BBB penetration following alcohol drinking^84^ may contribute to the ability of semaglutide to reach NAc. A role of GLP-1R in NAc in modulating alcohol response are provided by previous data demonstrating that local infusion of exendin-4 into NAcS decreases alcohol consumption in rats of both sexes.^31^^,^^85^ Moreover, the NAc expression of the *GLP-1R* gene is elevated in high-compared to low-alcohol preferring rats.^31^ Although it remains to be demonstrated in upcoming studies, we propose that semaglutide acts within the NAc to enhance dopamine metabolism which in turn suppresses alcohol-induced reward and decreases alcohol drinking.

While dopamine in the NAc appears to play a crucial role in the interaction between alcohol and semaglutide, other reward-related areas such as the amygdala^86^^,^^87^ may also be of interest. It is worth noting that semaglutide tended to increase cAMP in this region in alcohol naive mice. Unlike the lateral septum, the laterodorsal tegmental area and paraventricular thalamus, which showed altered monoaminergic neurotransmission in alcohol-drinking rats, may also be of interest for the understanding semaglutide's mechanisms of action.

In addition to the observed reduction in alcohol intake, semaglutide dose-dependently decreased chow intake and reduced body weight in alcohol-drinking male and female rats. Moreover, semaglutide reduced the total caloric intake in alcohol-naïve male mice. Supportively, preclinical and clinical studies show an anorexigenic effect by higher semaglutide doses^21^^,^^35^^,^^88^^,^^89^ and by other GLP-1R agonists.^15^^,^^90^, ^91^, ^92^ Moreover, a body weight reduction is evident by semaglutide, dulaglutide, liraglutide and exendin-4 in obese rodents^35^^,^^88^^,^^93^^,^^94^ and by dulaglutide and liraglutide in alcohol-drinking rats.^17^^,^^23^ While 0.026 mg/kg of semaglutide reduced feeding similarly in both sexes, the dose of 0.052 mg/kg had a more profound effect in males. Moreover, the reduction in body weight by both semaglutide doses was more pronounced in males. The mechanisms behind the observed sex differences in response to GLP-1R agonists remain unclear, but could be related to sex hormones. Indeed, oestrogen has been shown to influence the response to these agonists in feeding studies.^95^^,^^96^ Moreover, pharmacokinetic differences and sex-specific molecular mechanisms like monoamines in the VTA may also play important roles.

The findings that semaglutide reduced alcohol and food intake, lowered body weight in both male and female rats, and decreased the intake of rewarding foods in male mice, collectively indicate that semaglutide has an overall suppressive effect on motivation for rewards. On a similar note, other GLP-1R agonists have been shown to attenuate the responses to addictive drugs including nicotine, cocaine and opioids (for review see^97^). Moreover, activation of GLP-1R has been shown to suppress the reward value associated with food and reduce the motivation to acquire both ordinary chow and highly rewarding food items.^98^ The impact of GLP-1 on the overall motivation for rewards appears to extend to other gut-brain peptides. For instance, ghrelin has been shown to enhance responses to alcohol, drugs of abuse and foods.^97^^,^^98^

It is worth noting that semaglutide exhibits a differential effect on water consumption based on sex. Specifically, an increase in water intake was observed in females, whereas males showed no change or a decrease in water intake. On a similar note, other GLP-1R agonists increase water intake in males. Whether this elevation in females is a consequence of the decreased alcohol intake or depends on neurobiological underpinnings remains unknown.^15^, ^16^, ^17^ It is worth considering the potential involvement of vasopressin in the observed increase in water intake since GLP-1R signalling inhibits the production of this hormone.^99^ Another tentative factor that may contribute to the increased water intake observed with semaglutide is GLP-1's ability to enhance sodium excretion.^100^ Moreover, the neuronal mechanisms contributing towards the sex-divergent effects is most likely complex, but may involve factors such as differing sensitivity of osmoreceptors in the hypothalamus or variations in vasopressin levels between the sexes.^101^

In conclusion, semaglutide was found to decrease alcohol intake and reduce relapse-like drinking in rats of both sexes. By blocking the alcohol-induced hyperlocomotion and dopamine elevation in the NAc, semaglutide may attenuate alcohol reward and consequently suppress alcohol consumption. These findings further suggest that semaglutide could be a promising treatment for AUD, particularly in overweight patients, with a potential mechanism of action involving dopamine metabolism in the NAc.

## Contributors

CA designed the study, conducted hands-on work, analysed data, managed literature search, and wrote the first draft of manuscript. CEE performed parts of the hands-on work, and developed methodology. OTS, QZ, SBS, SW, MTA, LZ and DV performed parts of the hands-on work. EJ designed the study, managed literature search, analysed data, wrote the manuscript and was responsible for funding investigation and project administration. All authors contributed to the conception, interpretation, writing the manuscript, figure outline/design, met criteria for authorship and approved the final manuscript. Underlying data from OTS, QZ, SBS, SW, MTA, LZ and DV was after initially access/verification by experimenter also accessed and verified by CA and EJH. Underlying data from CA was after initial access/verification by experimenter also accessed and verified by EJH and OTS or SW. All authors had access to all data.

## Data sharing statement

Data collected for the study will be made available and shared to others though contact to the following address: elisabet.jerlhag@pharm.gu.se. The data will be share with anyone who wants to do additional analysis of the data and therefore the data will be shared after approval of a proposal, and with a signed data access agreement.

## Declaration of interests

EJ has secured funding for the current project, with the support being facilitated by the University. Additionally, EJ has authored a book chapter and consequently received royalties. These financial considerations have had no bearing on the project including the design of the experiments, the analysis and interpretation of the data, and the writing of the manuscript. The remaining authors declare no conflict of interest.
