# Supplementary material for: Semaglutide reduces alcohol intake and relapse-like drinking in male and female rats
Source: eBioMedicine. 2023 Jun 7;93:104642. doi: 10.1016/j.ebiom.2023.104642 (PMC10363436; doi:10.1016/j.ebiom.2023.104642)
Supplement: Supplementary Figs. S1–S15 [file mmc1.pdf]

Supplementary Figure 1

A.

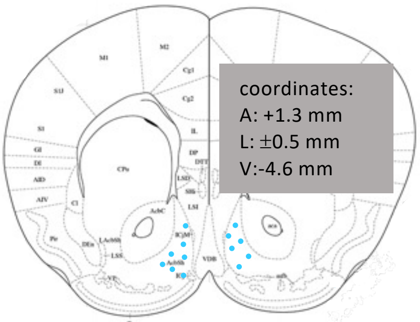

B.

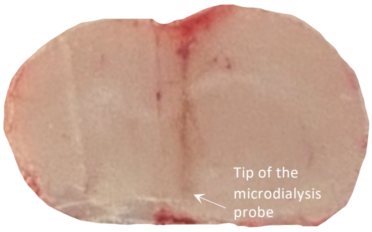

(A) Schematic illustration of 13 probe placements (illustrated by blue dots) in nucleus accumbens shell of male mice. (B) A representative placement of a microdialysis probe targeting nucleus accumbens shell, where the tip of the probe is highlighted with an arrow

Supplementary Figure 2

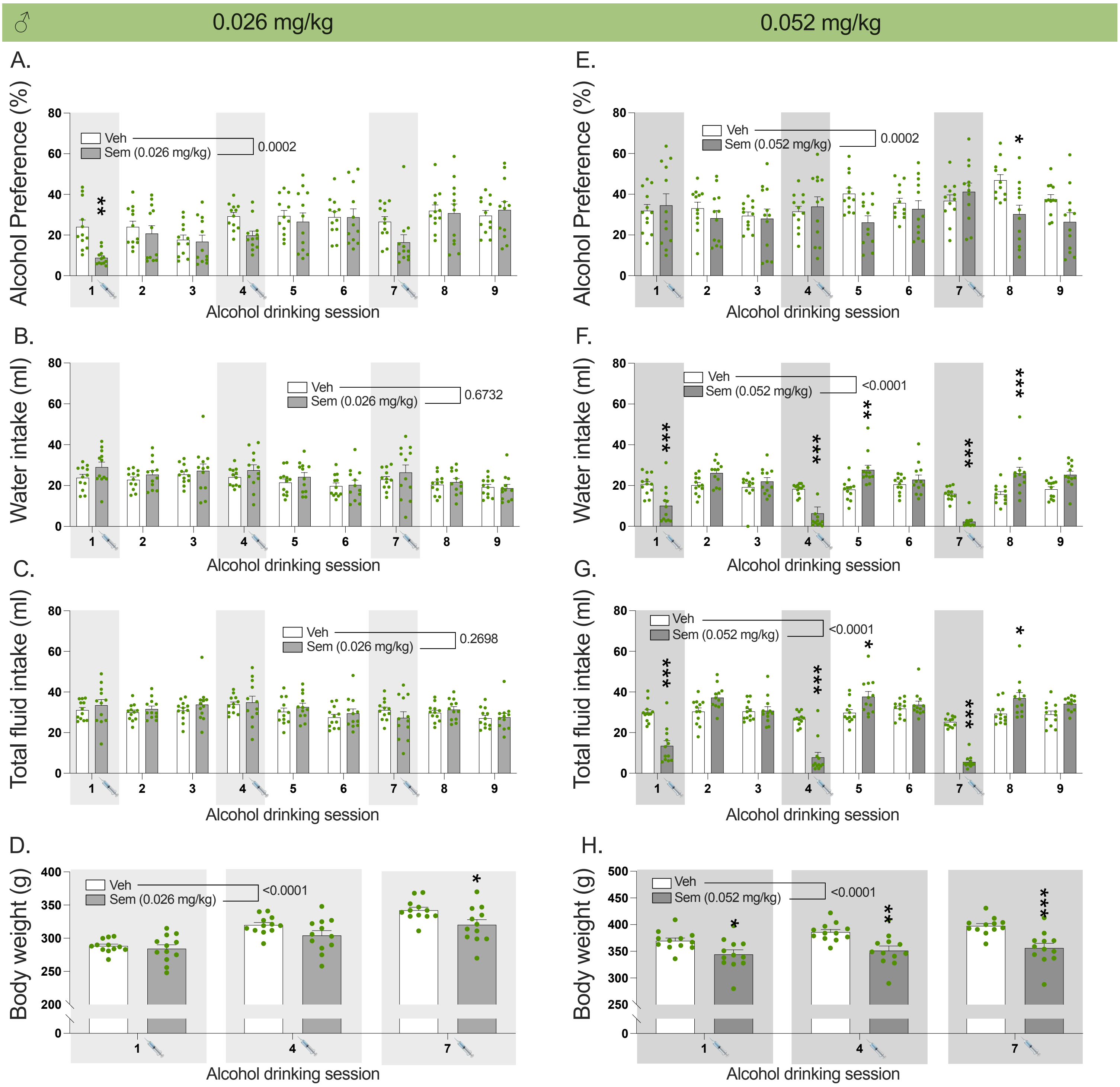

I.

Parameters of drinking experiments after semaglutide treatment					
Sex	*Parameter*	*Statistical analysis*			*Posthoc test*
Males, semaglutide 0.026 mg/kg	Alcohol preference	Repeated two-way ANOVA	time	F(8,176)=14.88 P<0.0001	P<0.01, S1
			treatment	F(1,22)=1.57 P=0.2228	
			interaction	F(8,176)=4.03, P=0.0002	
	Water intake		time	F(8,176)=7.67 P<0.0001	
			treatment	F(1,22)=1.03 P=0.3206	
			interaction	F(8,176)=0.72 P=0.6732	
	Total fluid intake		time	F(8,176)=5.87 P<0.0001	
			treatment	F(1,22)=0.30 P=0.5919	
			interaction	F(8,176)=1.26, P=0.2698	
	Body weight		time	F(2,44)=21.33 P<0.0001	P<0.05, S7
			treatment	F(1,22)=3.110 P=0.0917	
			interaction	F(2,44)=21.33, P<0.0001	
Males, semaglutide 0.052 mg/kg	Alcohol preference	Repeated two-way ANOVA	time	F(8,176)=3.03 P=0.0033	P<0.05, S8
			treatment	F(1,22)=1.60 P=0.2190	
			interaction	F(8,176)=4.01, P=0.0002	
	Water intake		time	F(8,176)=21.55 P<0.0001	P<0.01, S5<br>P<0.001, S1, S4, S7, S8
			treatment	F(1,22)=0.025 P=0.8737	
			interaction	F(8,176)=19.12, P<0.0001	
	Total fluid intake		time	F(8,176)=54.16 P<0.0001	P<0.05, S5, S8<br>P<0.001, S1, S4, S7
			treatment	F(1,22)=3.11 P=0.0916	
			interaction	F(8,176)=34.52, P<0.0001	
	Body weight		time	F(2,44)=224.10 P<0.0001	P<0.05, S1<br>P<0.01, S4<br>P<0.001, S7
			treatment	F(1,22)=11.74 P=0.0024	
			interaction	F(2,44)=36.06, P<0.0001	

In male rats, 0.026 mg/kg of semaglutide (A) alters alcohol preference, without changing (B) water or (C) total fluid intake. Semaglutide decreases (D) body weight. A dose of 0.052 mg/kg semaglutide (E) lowers alcohol preference, (F) water intake, (G) total fluid intake, and (H) body weight in male rats. (I) Statistical summary of the presented data. The overall interaction effect from the repeated two-way ANOVA is stated in the figure. Syringe indicates treatment with Vehicle (Veh), semaglutide (Sem).

Data are presented as mean ± SEM, significant data are illustrated by *P<0.05, **P<0.01, ***P<0.001.

Supplementary Figure 3

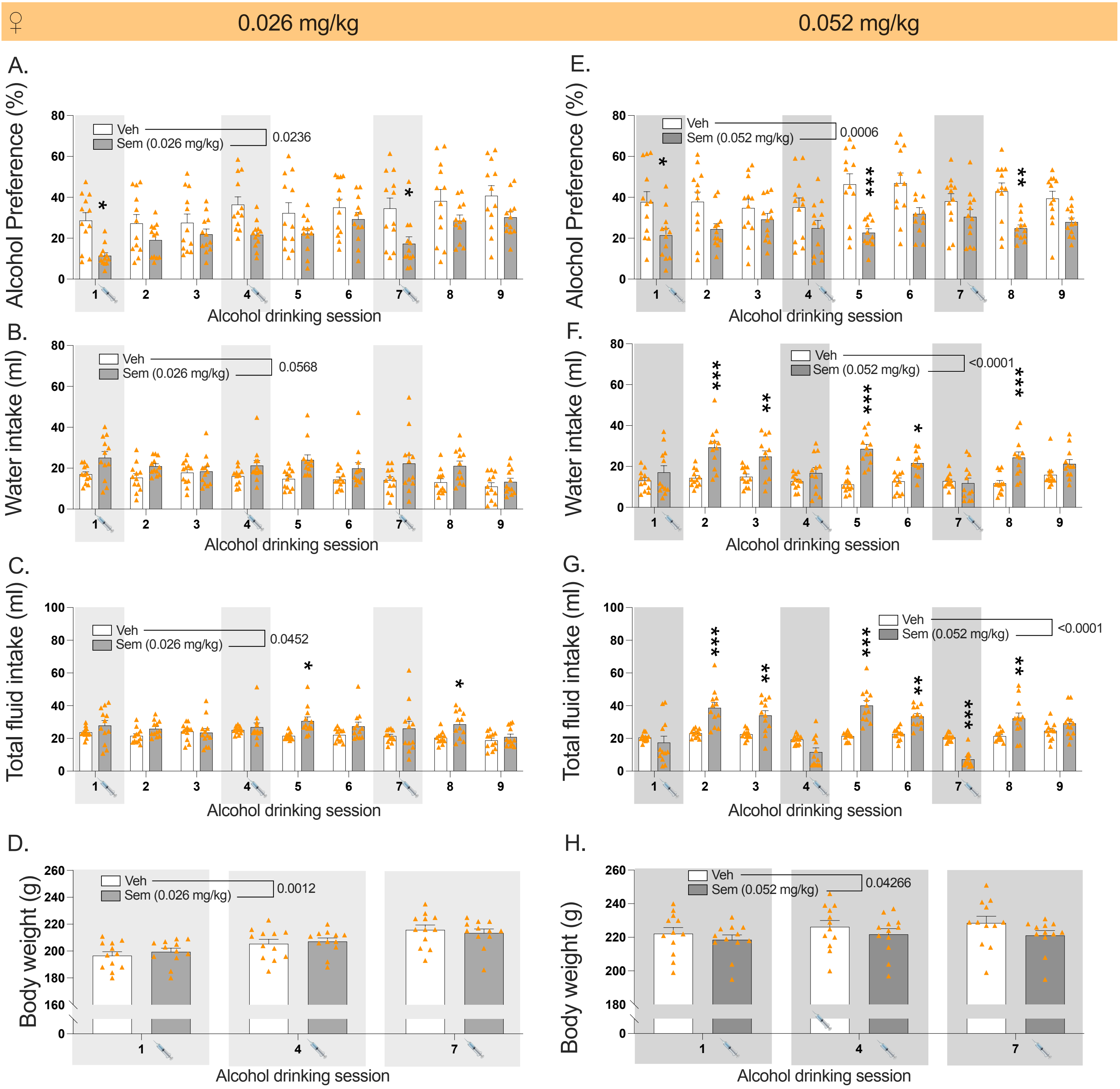

1.

Parameters of drinking experiments after semaglutide treatment					
Sex	*Parameter*	*Statistical analysis*			*Posthoc test*
Females, semaglutide 0.026 mg/kg	Alcohol preference	Repeated two-way ANOVA	time	F(8,176)=11.85 P<0.0001	P<0.05, S1, S7
			treatment	F(1,22)=5.89 P=0.0239	
			interaction	F(8,176)=2.29, P=0.0236	
	Water intake		time	F(8,176)=5.45 P<0.0001	n.s.
			treatment	F(1,22)=6.14 P=0.0214	
			interaction	F(8,176)=1.94, P=0.0568	
	Total fluid intake		time	F(8,176)=3.15 P=0.0023	P<0.05, S5, S8
			treatment	F(1,22)=4.37 P=0.0483	
			interaction	F(8,176)=2.03, P=0.0452	
	Body weight		time	F(2,44)=294.6 P<0.0001	n.s.
			treatment	F(1,22)=0.03 P=0.8666	
			interaction	F(2,44)=7.87, P=0.0012	
Females, semaglutide 0.052 mg/kg	Alcohol preference	Repeated two-way ANOVA	time	F(8,176)=4.20 P=0.0001	P<0.05, S1 P<0.01, S8 P<0.001, S5
			treatment	F(1,22)=8.52 P=0.0079	
			interaction	F(8,176)=3.62, P=0.0006	
	Water intake		time	F(8,176)=6.08 P<0.0001	P<0.05, S6 P<0.01, S3 P<0.001, S2, S5, S8
			treatment	F(1,22)=20.98 P=0.0001	
			interaction	F(8,176)=5.89, P<0.0001	
	Total fluid intake		time	F(8,176)=27.09 P<0.0001	P<0.01, S3, S6, S8 P<0.001, S2, S5, S7
			treatment	F(1,22)=8.34 P=0.0085	
			interaction	F(8,176)=19.28, P<0.0001	
	Body weight		time	F(2,44)=19.37 P<0.0001	n.s..
			treatment	F(1,22)=1.13 P=0.2988	
			interaction	F(2,44)=3.39, P=0.0426	

In female rats, 0.026 mg/kg of semaglutide (A) reduces alcohol preference, (B) without altering water and (C) increases the total fluid intake. (D) Semaglutide causes an overall decline in body weight. A dose of 0.052 mg/kg semaglutide (E) lowers alcohol preference, (F) increases water intake, and (G) total fluid intake. Moreover, (H) it has an overall reduction on body weight in female rats. (I) Statistical summary of the presented data.
The overall interaction effect from the repeated two-way ANOVA is stated in the figure. Syringe indicates time of injection of Vehicle (Veh) or semaglutide (Sem).

Data are presented as mean ± SEM, significant data are illustrated by *P<0.05, **P<0.01, ***P<0.001.

Supplementary Figure 4

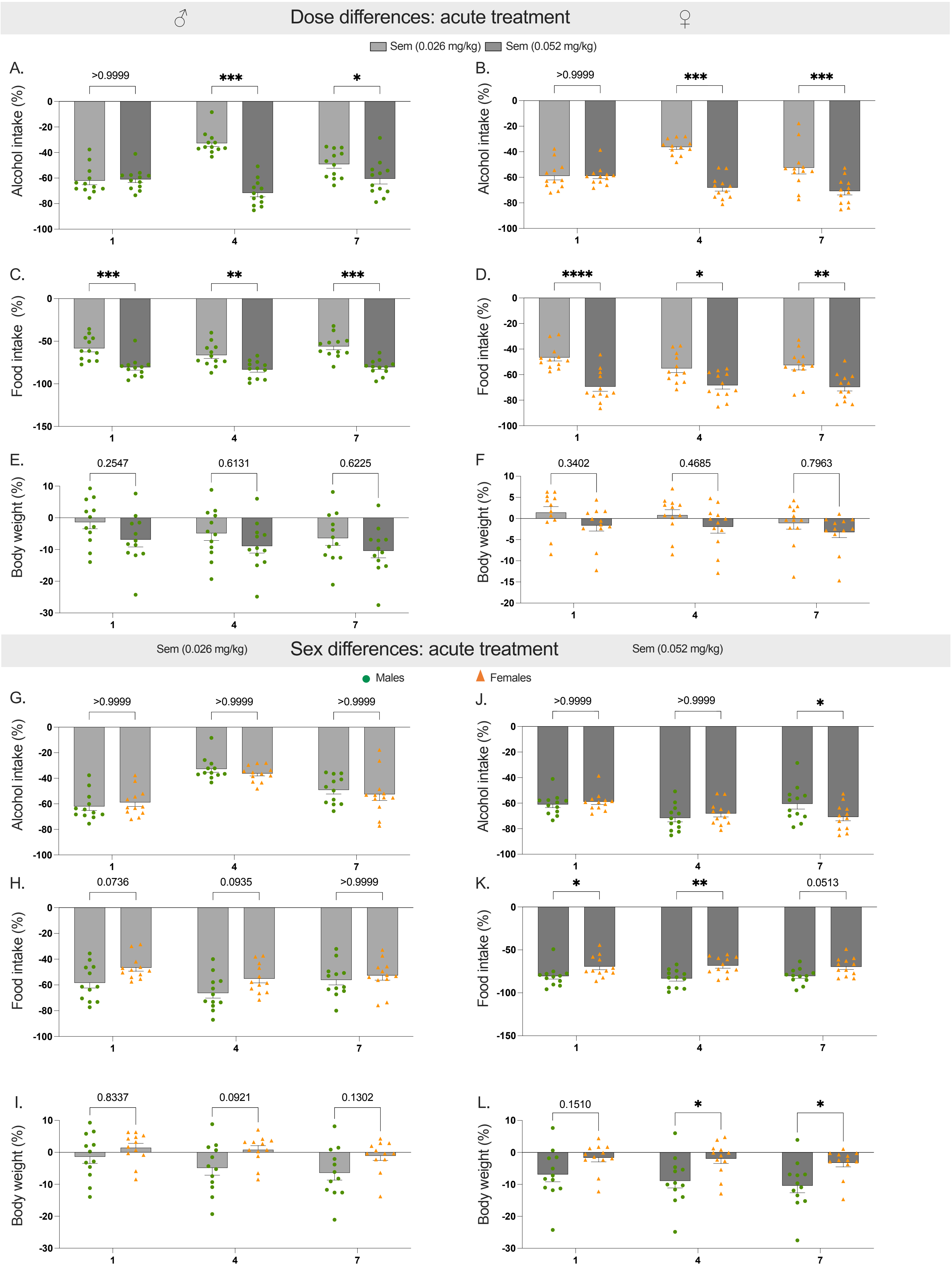

M.

Sex and dose differences after acute semaglutide treatment in alcohol drinking rats			
Comparision	*Sex or dose*	*Statistical analysis*	
Dose-response effects on alcohol intake	Males	time	F(2,44)=6.48, P=0.0034
		treatment	F(1,22)=28.18, P<0.0001
		interaction	F(2,44)=28.96, P<0.0001
	Females	time	F(2,44)=6.15, P=0.0004
		treatment	F(1,22)=31.40, P<0.0001
		interaction	F(2,44)=16.69, P<0.0001
Dose-response effects on food intake	Males	time	F(2,44)=11.59, P<0.0001
		treatment	F(1,22)=20.62, P=0.0002
		interaction	F(2,44)=3.47, P=0.0400
	Female	time	F(2,44)=1.14, P=0.3300
		treatment	F(1,22)=27.18, P<0.0001
		interaction	F(2,44)=1.71, P=0.1928
Dose-response effects on body weight	Males	time	F(2,44), 71.25, P<0.001
		treatment	F(1,22)=2.10, P=0.1618
		interaction	F(2,44)=2.77, P=0.0737
	Female	time	F(1,44)=19.06, P<0.0001
		treatment	F(1,22)=2.01, P=0.1700
		interaction	F(2,44)=0.9040, P=0.4123
Sex-effects on alcohol intake	semaglutide 0.026 mg/kg	time	F(2,44)=40.69, P<0.0001
		treatment	F(1,22)=0.17, P=0.6818
		interaction	F(2,44)=0.87, P=0.4245
Sex-effects on food intake	semaglutide 0.026 mg/kg	time	F(2,44)=7.47, P=0.0016
		treatment	F(1,22)=4.01, P=0.0576
		interaction	F(2,44)=2.15, P=0.1287
Sex-effects on body weight	semaglutide 0.026 mg/kg	time	F82,44)=48.79, P<0.0001
		treatment	F(1,22)=3.29, P=0.0834
		interaction	F(2,44)=8.37, P=0.0008
Sex-effects on alcohol intake	semaglutide 0.052 mg/kg	time	F(2,44)=7.76, P=0.0013
		treatment	F(1,22)=0.26, P=0.6135
		interaction	F(2,44)=4.49, P=0.0168
Sex-effects on food intake	semaglutide 0.052 mg/kg	time	F(2,44)=0.10, P=0.9085
		treatment	F(1,22)=10.69, P=0.0035
		interaction	F(2,44)=0.68, P=0.5107
Sex-effects on body weight	semaglutide 0.052 mg/kg	time	F(2,44)=30.39, P<0.0001
		treatment	F(1,22)=6.18, P=0.0210
		interaction	F(2,44)=5.24, P=0.0091

In both (A) male and (B) female rats, 0.052 mg/kg of semaglutide reduces alcohol intake more than 0.026 mg/kg. This dose-response effect is also evident when it comes to food intake in both (C) male and (D) female rats. Contrarily, the ability of both doses to reduce body weight is similar in (E) males and (F) females.

The dose of 0.026 mg/kg reduces (G) alcohol intake and (H) food intake with the same magnitude in both sexes. (I) There is an overall dose-response effect on body weight between sexes, where the reduction tends to be higher in male rats. The dose of 0.052 mg/kg (J) reduces alcohol intake more in females than in males. Contrarily, 0.052 mg/kg, decreases (K) food intake and (L) body weight to a greater extent in males compared to females. Semaglutide (Sem).

Data are presented as mean ± SEM over each of the three acute treatment sessions where the rats also were drinking alcohol (session 1, 4 7). Each parameter is shown as a % reduction, in males (green circles) and females (orange triangles). Or % reduction by 0.026 mg/kg (light grey) or 0.052 mg/kg (dark grey).

Significant data are illustrated by *P<0.05, **P<0.01, ***P<0.001.

Supplementary Figure 5

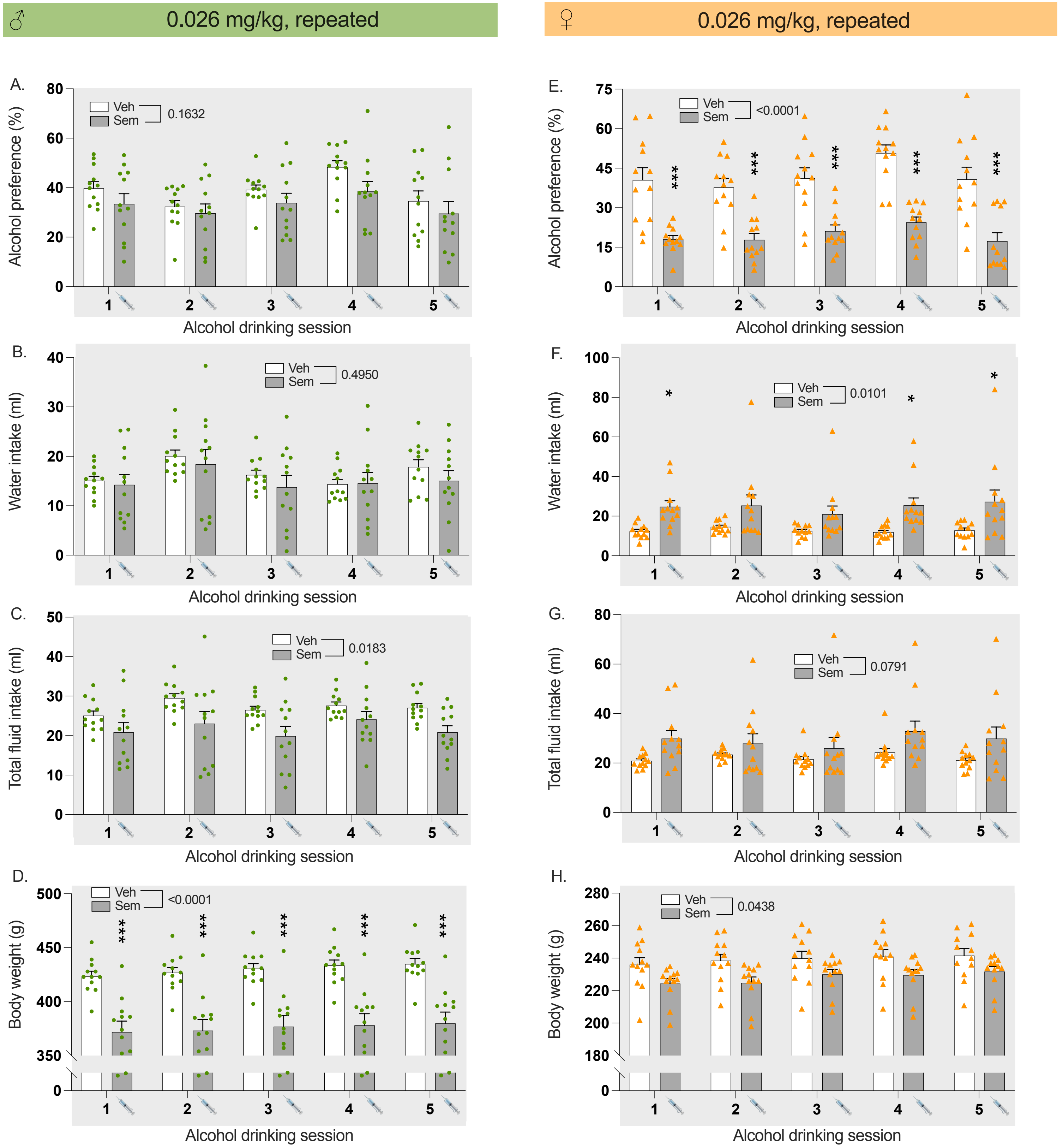

I.

Parameters of drinking experiments after repeated semaglutide treatment in alcohol drinking male and female rats					
Sex	*Parameter*	*Statistical analysis*			*Posthoc test*
Males, semaglutide 0.026 mg/kg	Alcohol preference	Repeated two-way ANOVA	time	F(4,88)=9.04 P<0.0001	
			treatment	F(1,22)=2.08 P=0.1632	
			interaction	F(4,88)=0.65, P=0.6286	
	Water intake		time	F(4,88)=5.83 P=0.0003	
			treatment	F(1,22)=0.48 P=0.4950	
			interaction	F(4,88)=0.55, P=0.7030	
	Total fluid intake		time	F(4,88)=3.36 P=0.0132	
			treatment	F(1,22)=6.50 P=0.0183	
			interaction	F(4,88)=0.75, P=0.5576	
	Body weight		time	F(4,88)=46.36 P<0.0001	P<0.0001, S1, S2, S3, S4, S5
			treatment	F(1,22)=22.14 P=0.0001	
			interaction	F(4,88)=1.61, P=0.1799	
Femles, semaglutide 0.026 mg/kg	Alcohol preference	Repeated two-way ANOVA	time	F(4,88)=11.44 P<0.0001	P<0.0001, S1, S2, S3, S4, S5
			treatment	F(1,22)=28.95 P<0.0001	
			interaction	F(4,88)=1.35, P=0.2570	
	Water intake		time	F(4,88)=1.55 P=0.1957	P<0.05, S1, S4, S5
			treatment	F(1,22)=7.93 P=0.0101	
			interaction	F(4,88)=1.19, P=0.3201	
	Total fluid intake		time	F(4,88)=3.35 P=0.0136	
			treatment	F(1,22)=3.39 P=0.0791	
			interaction	F(4,88)=1.53, P=0.2014	
	Body weight		time	F(4,88)=48.95 P<0.0001	
			treatment	F(1,22)=4.58 P=0.0438	
			interaction	F(4,88)=3.91, P=0.0058	

In male rats, repeated semaglutide treatment (A) does not alter the alcohol preference or (B) water intake. (C) It has an overall reduction on total fluid intake. Repeated treatment with semaglutide decreases (D) body weight. In female rats, repeated semaglutide treatment (E) lowers alcohol preference, (F) increases water intake, (G) without altering the total fluid intake. Moreover, repeated treatment with semaglutide (H) has an overall reduction on body weight. (I) Statistical summary of the presented data. The overall treatment effect from the repeated two-way ANOVA is stated in the figure. Syringe indicates time of injection of Vehicle (Veh) or semaglutide (Sem).

Data are presented as mean ± SEM, significant data are illustrated by *P<0.05, **P<0.01, ***P<0.001.

Supplementary Figure 6

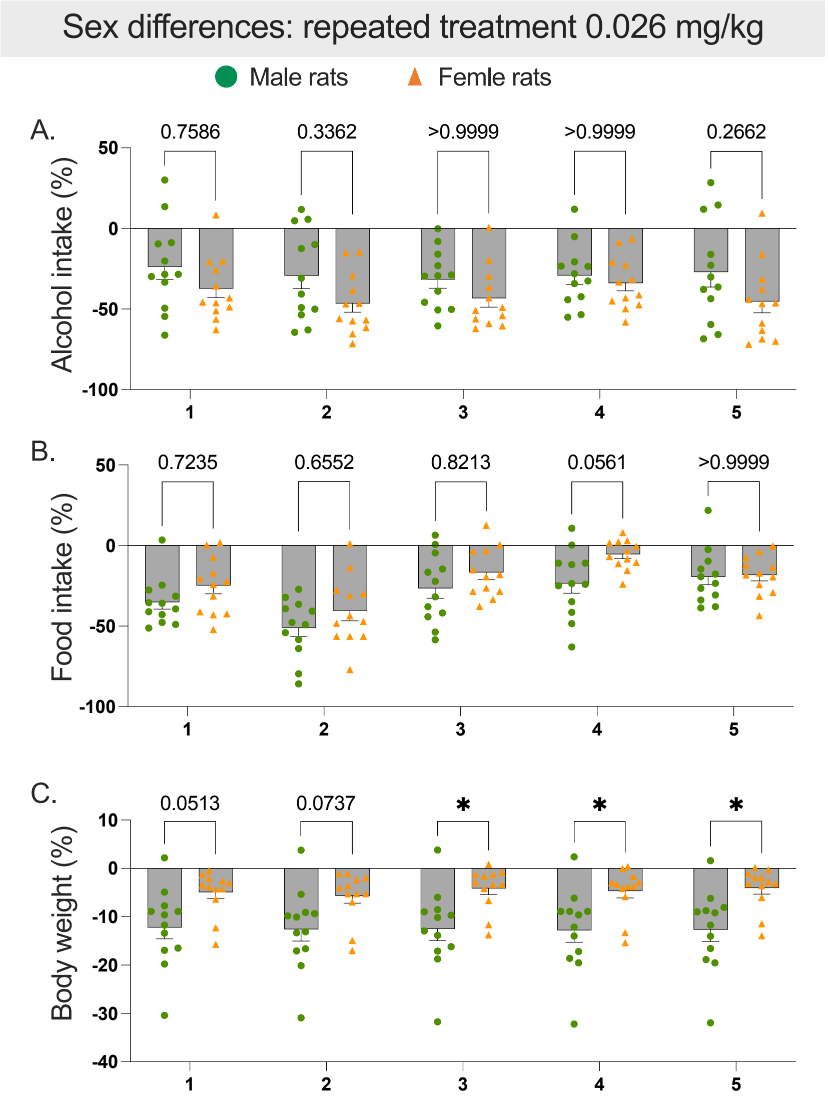

D.

Sex differences in alcohol drinking rats treated repeatedly wtih semaglutide			
Parameter	*Statistical analysis*		
Alcohol intake	Repeated two-way ANOVA	time	F(4,88)=1.09, P=0.3657
		treatment	F(1,22)=3.27, P=0.0843
		interaction	F(4,88)=0.66, P=0.6203
Food intake		time	F(4,88)=31.88, P<0.0001
		treatment	F(1,22)=2.94, P=0.1005
		interaction	F(4,88)=1.92, P=0.1150
Body weight		time	F(4,88)=4.51, P=0.0023
		treatment	F(1,22)=8.04, P=0.0096
		interaction	F(4,88)=5.50, P=0.0005

The ability of repeated semaglutide (0.026 m/kg) treatment to decrease (A) alcohol drinking or (B) food intake, is similar between alcohol drinking rats of both sexes. On the contrary, (C) the body weight reduction is more evident in male rats compared to female rats. Vehicle (Veh), semaglutide (Sem). Each parameter is shown as a % reduction, in males (green circles) and females (orange triangles).

Data are presented as mean ± SEM, over each of the alcohol drinking sessions where the rats also were treated with semaglutide (session 1-5). Significant data are illustrated by *P<0.05.

Supplementary Figure 7

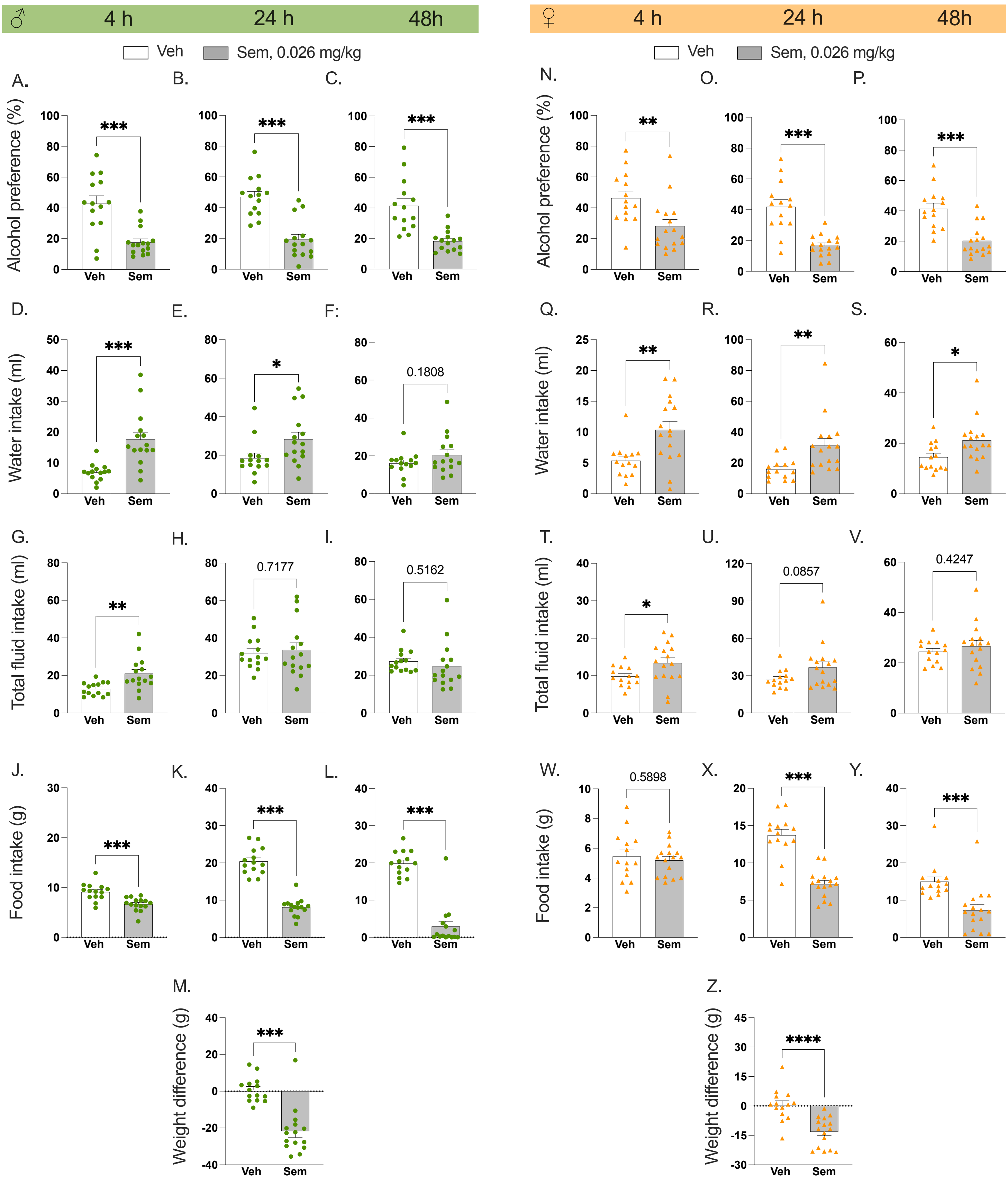

AB.

Effects of acute semaglutide treatment on parameteres after alcohol withdrawal in male and female rats			
Sex	*Parameter*	*Statistical analysis, upaired t-test*	
Male rats	Alcohol preference	4 h	t(27)=4.66, P<0.0001
		24 h	t(27)=5.85, P<0.0001
		48 h	t(27)=4.77, P<0.0001
	Water intake	4 h	t(27)=4.22, P=0.0002
		24 h	t(27)=2.23, P=0.0345
		48 h	t(27)=1.37, P=0.1808
	Total fluid intake	4 h	t(27)=3.25, P=0.0031
		24 h	t(27)=0.37, P=0.7177
		48 h	t(27)=0.66, P=0.5162
	Food intake	4 h	t(27)=4.32, P=0.0002
		24 h	t(27)=11.03, P<0.0001
		48 h	t(27)=9.90, P<0.0001
	Body weight	24 h	t(27)=5.88, P<0.0001
Femle rats	Alcohol preference	4 h	t(28)=2.96, P=0.0062
		24 h	t(28)=5.50, P<0.0001
		48 h	t(28)=4.92, P<0.0001
	Water intake	4 h	t(28)=3.15, P=0.0039
		24 h	t(28)=3.01, P=0.0055
		48 h	t(28)=2.55, P=0.0164
	Total fluid intake	4 h	t(28)=2.26, P=0.0322
		24 h	t(28)=1.78, P=0.0857
		48 h	t(28)=0.81, P=0.4247
	Food intake	4 h	t(28)=0.55, P=0.5853
		24 h	t(28)=7.64, P<0.0001
		48 h	t(28)=3.81, P=0.0007
	Body weight	24 h	t(28)=4.72, P<0.0001

In male rats, semaglutide decreases (A-C) alcohol preference at 4, 24, 48 hours (h). It increases water intake at 4 h and 24 h (D-F), as well as (G-I) elevates total fluid intake at 4h. Moreover, semagutide (J-L) decreases food intake at all time points and (M) lowers the body weight difference. In female rats, semaglutide (N-P) decreases alcohol preference and (Q-S) increases water intake at all time points. (T-V) Semaglutide increases total fluid intake at 4h. Moreover, semaglutide (W-Y) decreases food intake at 24 and 48 h and (Z) lowers body weight difference. (AB) Statistical summary of the results from the drinking experiment with repeated treatment. Vehicle (Veh), semaglutide (Sem).

Data are presented as mean ± SEM, significant data are illustrated by *P<0.05, **P<0.01, ***P<0.001.

Supplementary Figure 8

♂ Alcohol-induced locomotor stimulation in male mice

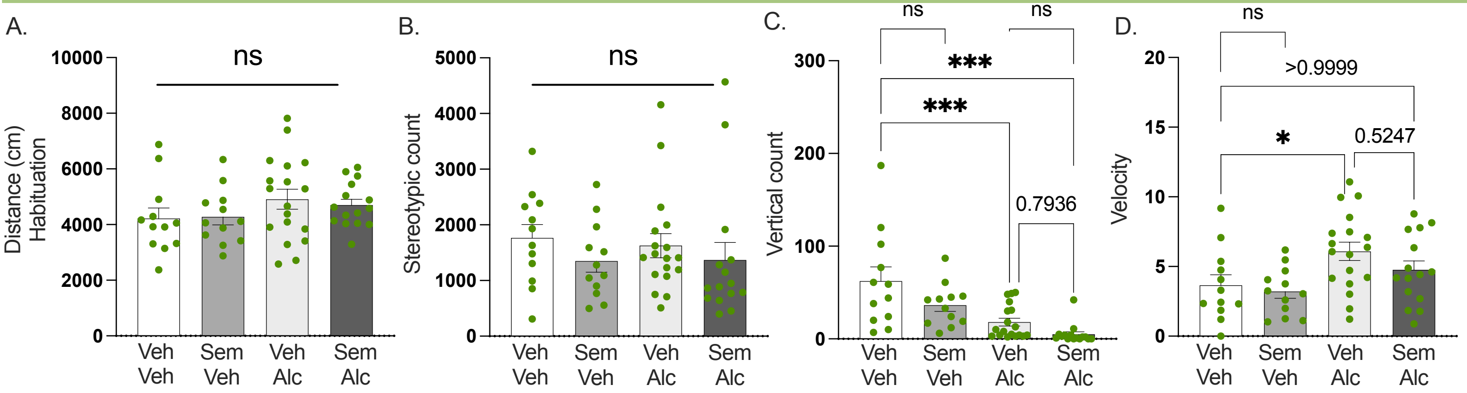

E.

Effects of acute semaglutide treatment on other locomotor activity paremeters in male mice treated with semaglutide and alcohol			
Sex	*Parameter*	*Statistical analysis, one-way ANOVA*	*Posthoc test*
Male mice	Distance during habitation	F (3,53)=2.28, P=0.0901	
	Stereotypic counts	F(3,53)=0.12, P=0.9451	
	Vertical counts	F(3,53)=10.03, P<0.0001	Veh-Veh vs Veh-Alc, P=0.0005<br>Veh-Veh vs Sem-Alc, P<0.0001<br>Veh-Alc vs Sem-Alc, P=0.7936
	Velocity	F(3, 53)=4.03, P=0.0118	Veh-Veh vs Veh-Alc, P=0.0445<br>Veh-Veh vs Sem-Alc, P=0.5247<br>Veh-Veh vs Sem-Alc, P>0.9999

(A) The ambulatory distance is similar between male mice during habituation, *i.e.* prior to treatment with vehicle (Veh), alcohol (Alc), semaglutide (Sem). (B) Neither alcohol or semaglutide treatment effects the stereotypic counts. (C) Compared to vehicle treatment, alcohol reduces the vertical counts. An effect not changed by semaglutide treatment. (D) Alcohol enhances the velocity, and this is unaffected by semaglutide treatment in male mice.

Data are presented as mean ± SEM, significant data are illustrated by *P<0.05, **P<0.01, ***P<0.001.

Supplementary Figure 9

♂ 4 hours rewarding food intake in alcohol naïve male mice

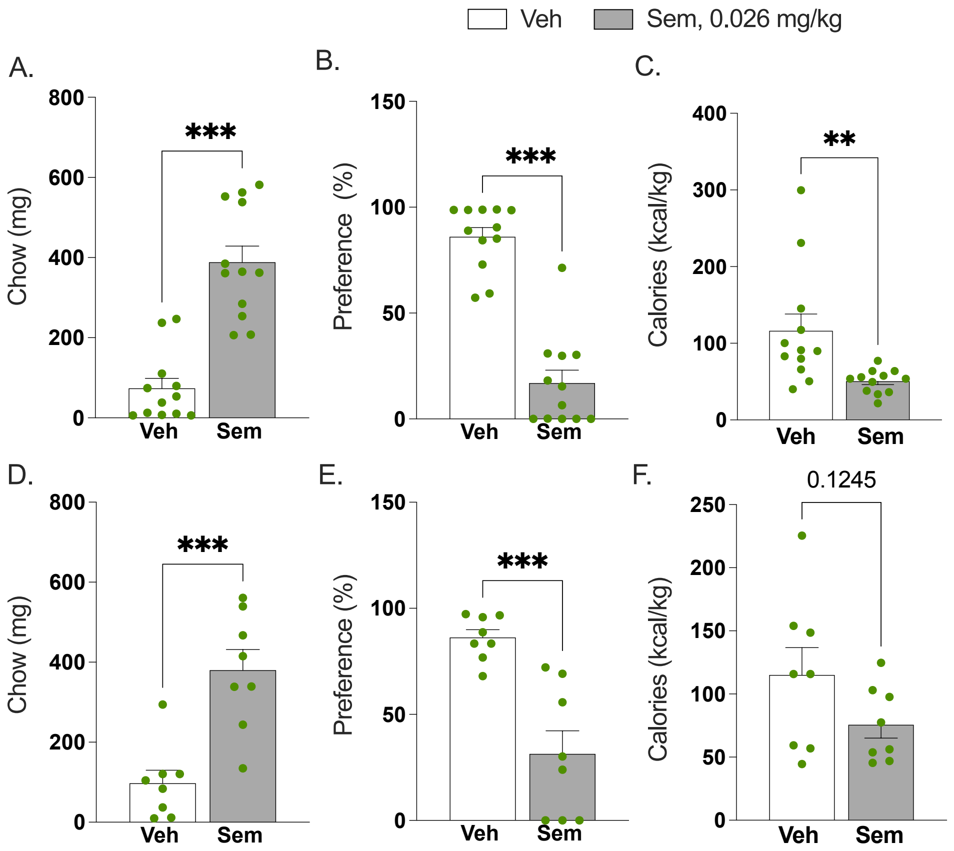

G.

Semaglutide-induced effects on additional feeding parameters at the four hour time point in male mice			
Sex	*Parameter*		*Statistical analysis, unpaired t-test*
Male mice, alcohol naïve	Intake of peanut butter, 4 hours	Chow	t(22)=6.66, P<0.0001
		Preference	t(22)=9.11, P<0.0001
		Caloric intake	t(22)=2.92, P=0.0079
	Intake of Nutella, 4 hours	Chow	t(14)=4.65, P=0.0004
		Preference	t(14)= 4.76, P=0.0003
		Caloric intake	t(14)=1.13, P=0.1245

Compared to vehicle (Veh, n=12), semaglutide (Sem, n=12) (A) increase chow intake, (B) declines the preference for peanut butter, (C) without altering the caloric intake in alcohol naïve male mice. Semaglutide (D) increases chow intake, (E) tends to reduce the preference for Nutella, (F) without altering the caloric intake (n=8 per treatment group). (G) Statistical summary of data obtained.

Data are presented as mean ± SEM, significant data are illustrated by **P<0.05, ***P<0.001.

Supplementary Figure 10

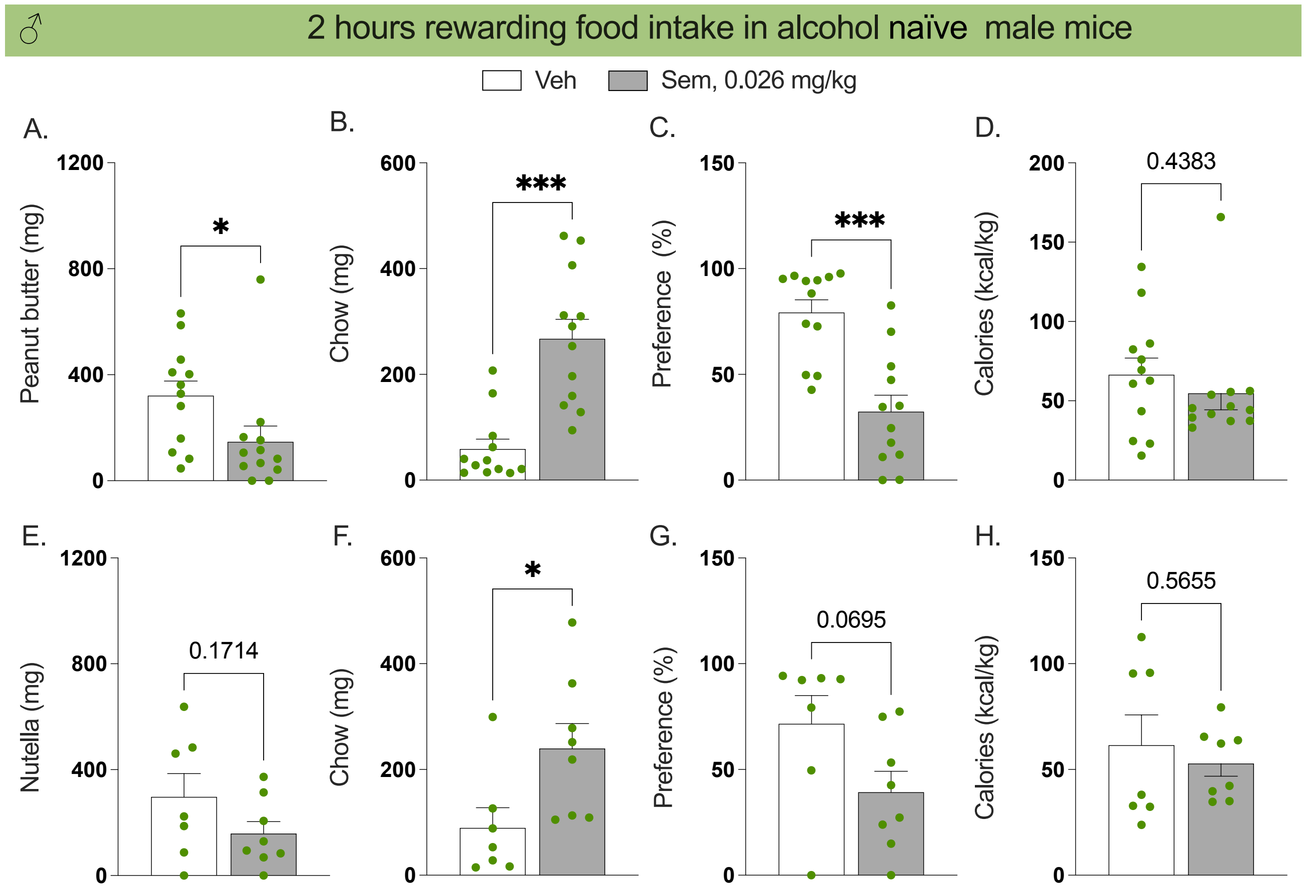

I.

Rewarding food intake after semaglutide treatment in alcohol naïve male mice			
Sex	*Parameter*		*Statistical analysis, unpaired t-test*
Male miace, alcohol naïve	Intake of peanut butter, 2 hours	Peanut butter	t(22)=2.15, P=0.0430
		Chow	t(22)=5.08, P<0.0001
		Preference	t(22)=4.76, P<0.0001
		Caloric intake	t(22)=0.79, P=0.4383
	Intake of Nutella, 2 hours	Nutella	t(13)=1.45, P=0.1714
		Chow	t(13) = 2.42, P=0.0309
		Preference	t(13)= 1.98, P=0.0695
		Caloric intake	t(13)=0.59, P=0.5655

(A) Compared to vehicle (Veh), semaglutide (Sem) (A) reduces the peanut butter intake, (B) increase chow intake, (C) declines the preference for peanut butter, (D) without altering the caloric intake in alcohol naïve male mice. Semaglutide (E) tends to decrease the intake of Nutella, (F) increases chow intake, (G) tend to reduce the preference for Nutella, (H) without altering the caloric intake in alcohol naïve male mice. (I) Statistical summary of all obtained data at the 2 hour time point.

Data are presented as mean ± SEM, significant data are illustrated by *P<0.05, ***P<0.001.

Supplementary Figure 11

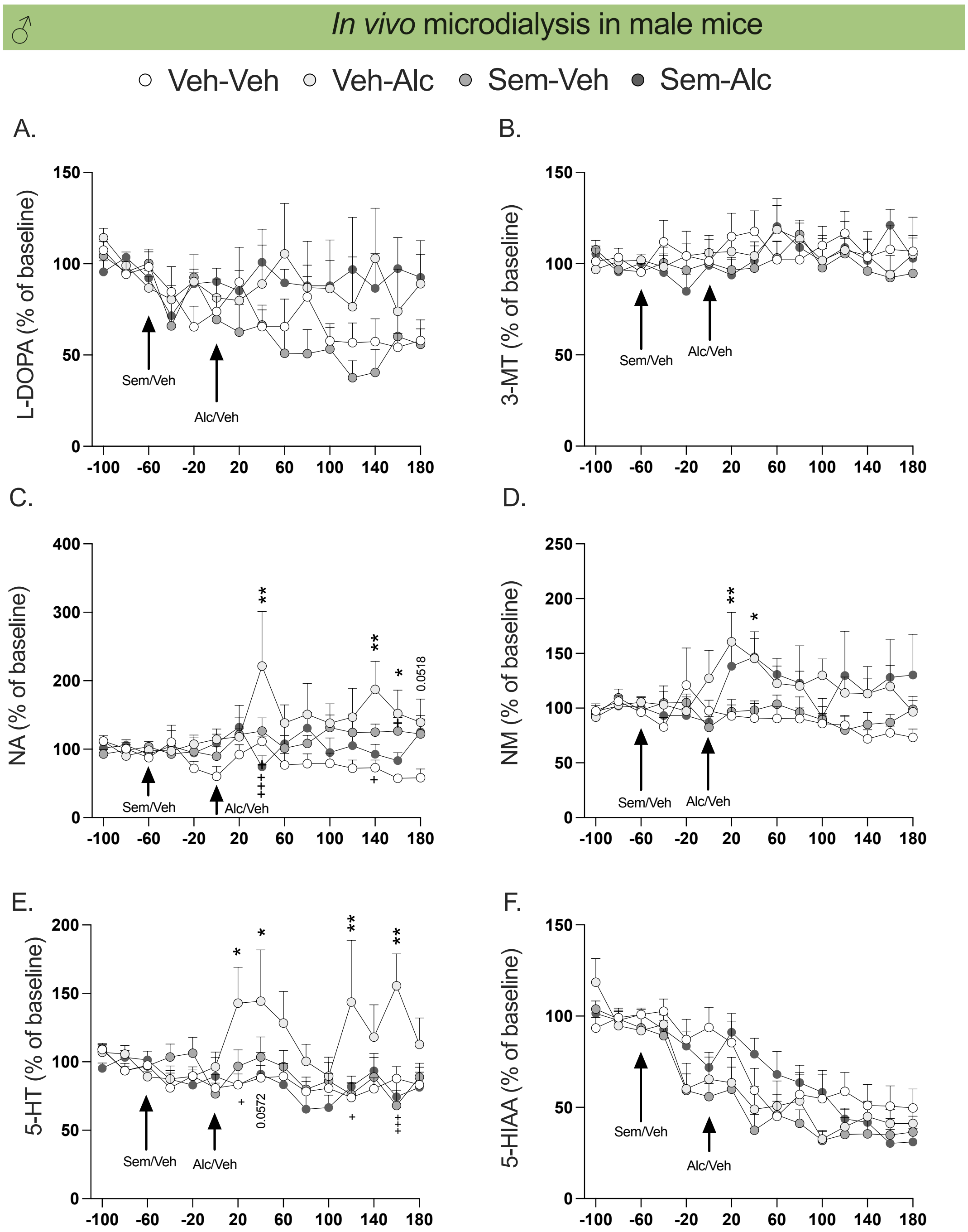

G.

In vivio monoaminergic neurotransmission in nucleus accumbens shell after alcohol and semaglutide treatment in male mice					
Sex	*Parameter*	*Statistical analysis, repeated two-way ANOVA*		*Posthoc test*<br><br>Veh-Veh<br>Vs<br>Veh-Alc	 <br>Veh-Alc<br>Vs<br>Sem-Alc
Male mice	L-DOPA	treatment	F(3,21)=1.93, P=0.1566		
		time	F(14,294)=3.42, P<0.0001		
		interaction	F(42,294)=1.17, P0.02275		
	3-MT	treatment	F(3,37)=0.3288, P=0.8045		
		time	F(14,518)=0.8468, P=0.6178		
		interaction	F(42,518)=0.5803, P=0.9844		
	NA	treatment	F(3,30)=3.37, P=0.0315	**40 min:**<br>P=0.0030<br>**140 min:**<br>P=0.0019<br>**160 min:**<br>P=0.0155<br>**180 min:**<br>P=0.0518	**40 min:**<br>P<0.0001<br>**140 min:**<br>P=0.0116
		time	F(14,420)=1.33, P=0.1867		
		interaction	F(42,420)=1.43, P=0.0449		
	NM	treatment	F(3,37)=3.26, P=0.0321	**20 min:**<br>P=0.0072<br>**40 min:**<br>P=0.0466	
		time	F(14,518)=1.32, P=0.1883		
		interaction	F(42,518)=1.15, P=0.2395		
	5-HT	treatment	F(3,37)=2.72, P=0.0582	**20 min:**<br>P=0.0159<br>**40 min:**<br>P=0.0279<br>**120 min:**<br>P=0.0030<br>**160 min:**<br>P=0.0043	**20 min:**<br>P=0.0251<br>**20 min:**<br>P=0.0572<br>**120 min:**<br>P=0.0188<br>**160 min:**<br>P=0.0008
		time	F(14,518)=1.48, P=0.1153		
		interaction	F(42,518)=1.45, P=0.0383		
	5-HIAA	treatment	F(3,33)=2.13, P=0.1147		
		time	F(14,462)=29.61, P<0.0001		
		interaction	F(42,462)=1.13, P=0.2713		

Obtained neurotransmission data from *in vivo* microdialysis of male mice treated with semaglutide (Sem), and alcohol (Alc) compared to vehicle (Veh).
(A) There is an overall interaction effect on L-DOPA release in nucleus accumbens shell, without any differences between treatments. (B) There are no overall effects on 3-MT release. (C) Alcohol increases noradrenalin (NA), and this is attenuated by semaglutide. (D) Alcohol also increases NM, an elevation also evident in semaglutide treated animals. (E) Alcohol enhances serotonin (5-HT), an effect attenuated by semaglutide. (F) There is no overall effect on 5-HIAA. (G) Statistical summary of *in vivo* microdialysis data.

Data are presented as mean ± SEM, significant data are illustrated by *P<0.05, **P<0.01 when comparing vehicle-vehicle to vehicle-alcohol. +P<0.05, ++P<0.001 when comparing vehicle-alcohol to semaglutide-alcohol.

Supplementary Figure 12

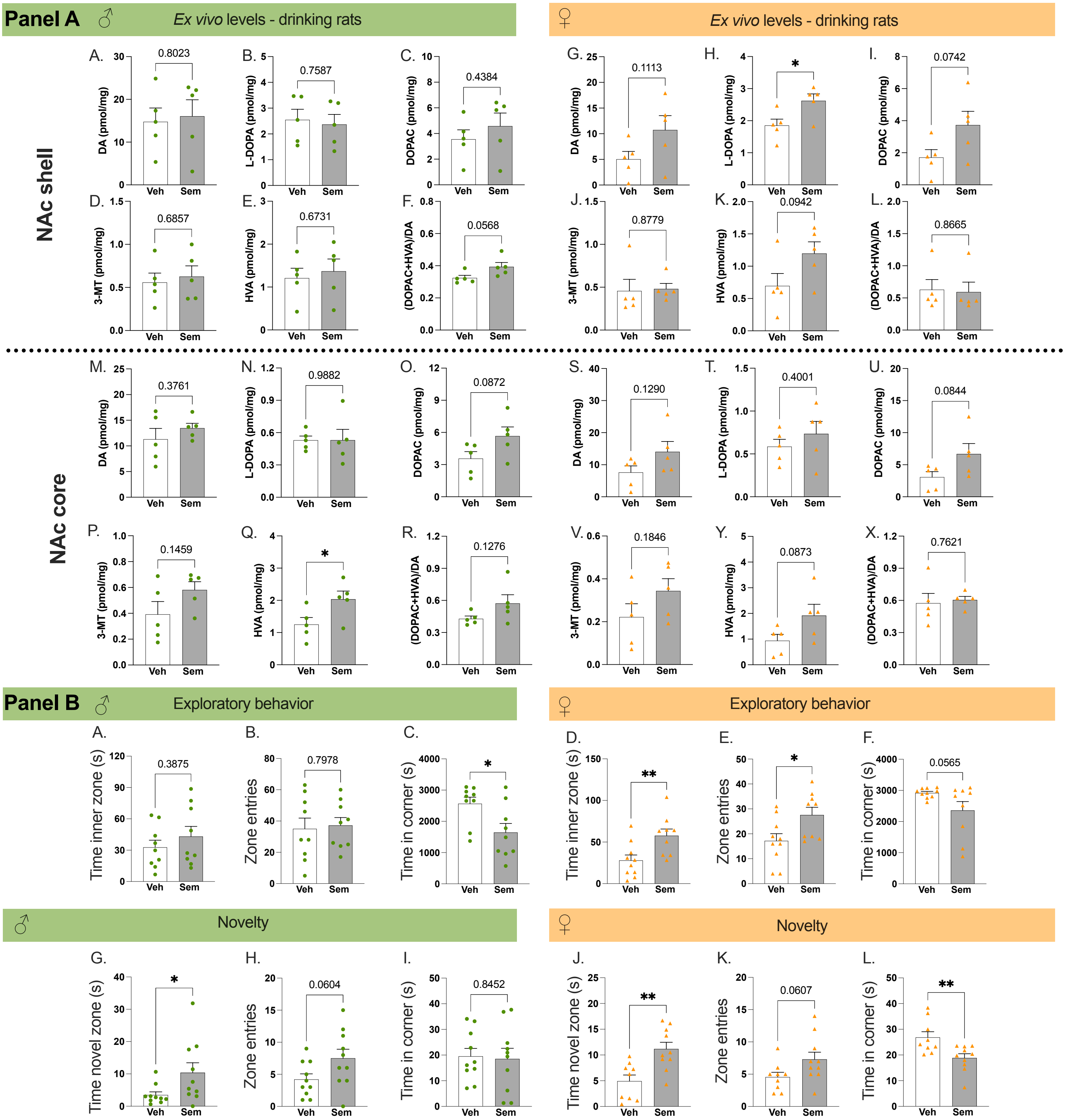

Semaglutide increases the *ex vivo* dopaminergic signaling in alcohol drinking male and female rats and enhances dopamine driven behaviors in alcohol naïve male and female rats				
Sex	*Experiment*	*Statistical analysis*		
Males	*Ex vivo* dopaminergic neurotransmission in nucleus accumbens shell	Unpaired t-test	DA	t(8)=0.26, P=0.8023
			L-Dopa	t(8)=0.32, P=0.7587
			DOPAC	t(8)=0.82, P=0.4384
			3-MT	t(8)=0.42, P=0.6857
			HVA	t(8)=0.44, P=0.6731
			(DOPAC+HVA)/DA	t(8)=2.23, P=0.0568
Females	*Ex vivo* dopaminergic neurotransmission in nucleus accumbens shell	Unpaired t-test	DA	t(8)=1.79, P=0.1113
			L-Dopa	t(8)=2.67, P=0.0285
			DOPAC	t(8)=2.05 P=0.0742
			3-MT	t(8)=0.16, P=0.8879
			HVA	t(8)=1.90, P=0.0942
			(DOPAC+HVA)/DA	t(8)=0.17 P=0.8665
Males	*Ex vivo* dopaminergic neurotransmission in nucleus accumbens core	Unpaired t-test	DA	t(8)=0.94, P=0.3761
			L-Dopa	t(8)=0.02, P=0.9982
			DOPAC	t(8)=1.95, P=0.0872
			3-MT	t(8)=1.61 P=0.1459
			HVA	t(8)=2.34 P=0.0476
			(DOPAC+HVA)/DA	t(8)=1.7, P=0.1276
Females	*Ex vivo* dopaminergic neurotransmission in nucleus accumbens core	Unpaired t-test	DA	t(8)=1.69, P=0.1290
			L-Dopa	t(8)=0.89, P=0.4001
			DOPAC	t(8)=1.97, P=0.0844
			3-MT	t(8)=1.45, P=0.1846
			HVA	t(8)=1.95, P=0.0873
			(DOPAC+HVA)/DA	t(8)=0.31, P=0.7621
Males	Exploratory behavior	Unpaired t-test	Time inner zone	t(16) = 0.89, P=0.3875
			Zone entries	t(16) = 0.26, P=0.7978
			Time in corner	t(16) = 2.60, P=0.0194
Female	Exploratory behavior	Unpaired t-test	Time inner zone	t(17) = 2.91, P=0.0098
			Zone entries	t(17) = 2.50, P=0.0249
			Time in corner	t(16) = 2.05, P=0.0565
Male	Novelty	Unpaired t-test	Time inner zone	t(18) = 2.14, P=0.0460
			Zone entries	t(18) = 2.00, P=0.0604
			Time in corner	t(18) = 0.20, P=0.8452
Female	Novelty	Unpaired t-test	Time inner zone	t(17) = 3.53, P=0.0026
			Zone entries	t(17) = 2.00, P=0.0607
			Time in corner	t(17) = 2.97, P=0.0086

Panel A: In alcohol drinking male rats, semaglutide does not alter ex vivo levels of (A) dopamine (DA), (B) L-DOPA, (C) DOPAC, (D) 3-MT, or (E) HVA, whereas it (F) tends to increase the dopamine turnover in NAc shell. In the same area of female alcohol drinking rats, semaglutide (G) does not affect DA (H), increases L-DOPA, (I) tends to increase DOPAC, (J) without changing 3-MT, (K) tends to enhance HVA (L) but not affect the dopamine turnover. In NAc core of alcohol drinking male rats, semaglutide does not affect the ex vivo levels of (M) DA or (N) L-LOPA, whereas it tends to increase (O) DOPAC and (P) 3-MT, (Q) elevates HVA and (R) tends to increase the dopamine turnover. In NAc core of female rats, semaglutide (S) tends to increase DA, (T) without affecting L-DOPA, tends to elevate (U) DOPAC, (V) 3-MT, (Y) HVA, (Z) without altering the dopamine turnover.

Panel B: In support for an interaction between semaglutide and dopamine signalling are the data from alcohol-naïve male and female rats showing that semaglutide enhances dopamine driven behaviours like exploratory behaviour and novelty seeking. In the exploratory test of male rats, semaglutide does not alter (A) the time in inner zone, or (B) zone entries, (C) whereas it decreases the time in corners. In female rats semaglutide (D) increases of the time in inner zone, (E) zone entries, and (F) subsequently tends to decrease the time in corners. Furthermore, semaglutide enhances novelty seeking in males as it (G) increases the time in novel zone, (H) tends to increase the zone entries, (I) without affecting the time in corners. Furthermore, semaglutide augments novelty seeking as it (J) increases the time in novel zone, (K) tends to increase the zone entries and (L) reduces the time in corners.

Data are presented as mean ± SEM, significant data are illustrated by *P<0.05, **P<0.01.

Supplementary Figure 13

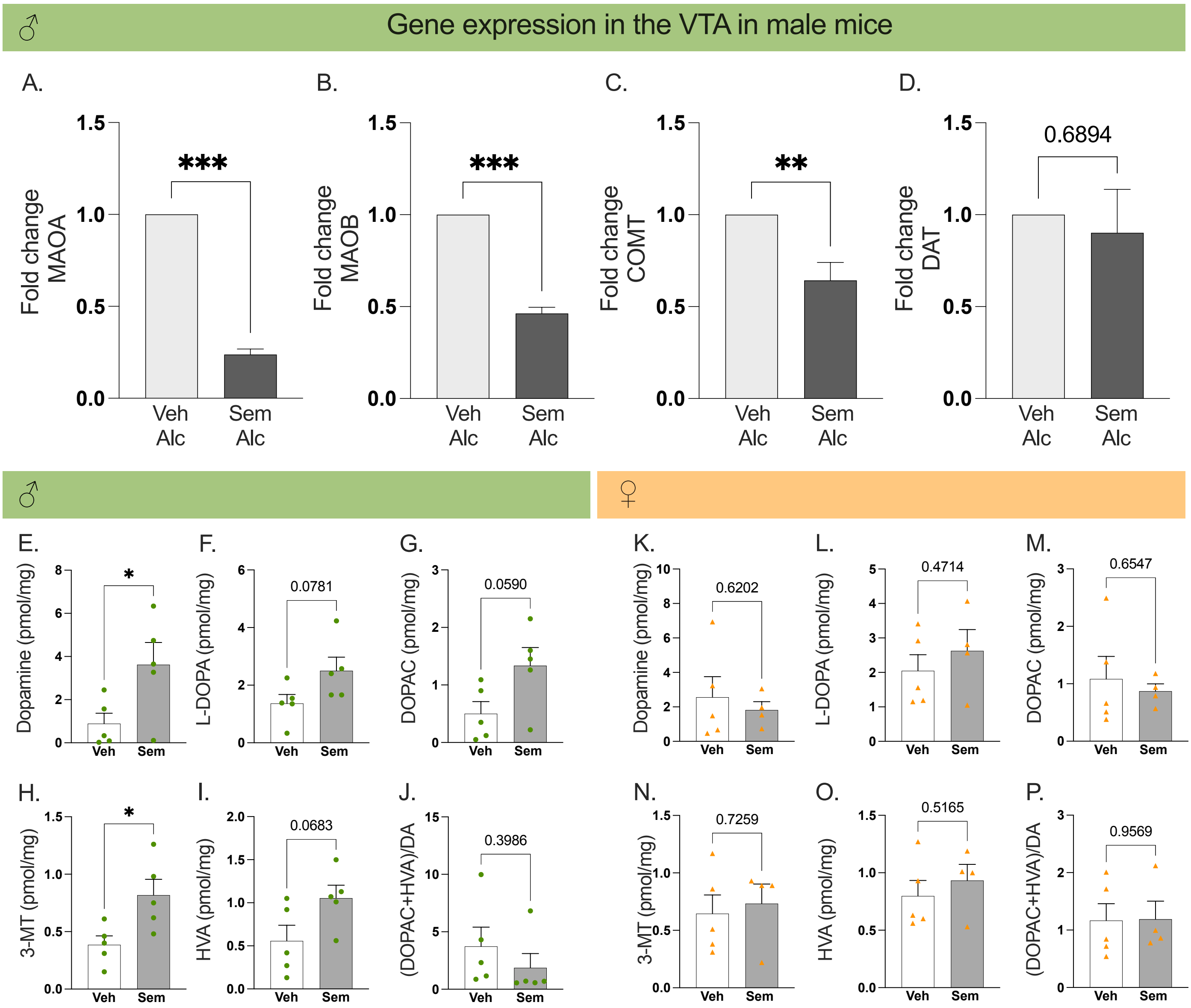

Q.

Effects on dopamine metabolism in the ventral tegmental area after semaglutide and alcohol treatment			
Sex	*Experiment*		*Statistical analysis, unpaired t-test*
Males	Gene expression after alcohol and semaglutide treatment	MAOA	t(8) = 25.47, P<0.0001
		MAOB	t(8) = 15.97, P<0.0001
		COMT	t(8) = 3.65, P=0.0065
		DAT	t(8) = 0.41, P=0.6894
	Ex vivo levels of monoaminergic neurotransmission after semaglutide treatment to alcohol drinking male rats	dopamine	t(8)=2.41, P=0.0426
		L-DOPA	t(8)=2.02, P=0.0781
		DOPAC	t(8)=2.20, P=0.0590
		3-MT	t(8)=2.74, P=0.0254
		HVA	t(8)=2.11, P=0.0683
		(DOPAC+HVA)/DA	t(8)=0.8917, P=0.3986
Females	Ex vivo levels of monoaminergic neurotransmission after semaglutide treatment to alcohol drinking female rats	Dopamine	t(7)=0.52, P=0.6202
		L-DOPA	t(7)=0.76, P=0.4714
		DOPAC	t(7)=0.47, P=0.6547
		3-MT	t(7),0.37, P=0.7259
		HVA	t(7)=0.68, P=0.5165
		(DOPAC+HVA)/DA	t(7)=0.06, P=0.9569

In comparison to alcohol, the combination of alcohol and semaglutide reduces the gene expression of (A) *MAOA*, (B) *MAOB*, (C) *COMT*, (D) without altering the gene expression of *DAT* in male mice. Analysis of the ventral tegmental area from male alcohol drinking rats reveals that the *ex vivo* levels of (E) dopamine are higher after semaglutide treatment. Treatment also tends to increase (F) L-DOPA, (G) DOPAC and (H) elevates 3-MT, (I) tends to enhance HVA, (J) without effecting the dopamine turnover. These differences are not evident in female alcohol drinking rats treated with semaglutide (Sem) or vehicle (Veh) (K-P). (Q) Statistical summary of the presented data.

Data are presented as mean ± SEM, significant data are illustrated by *P<0.05, **P<0.01 , ***P<0.001.

Supplementary Figure 14

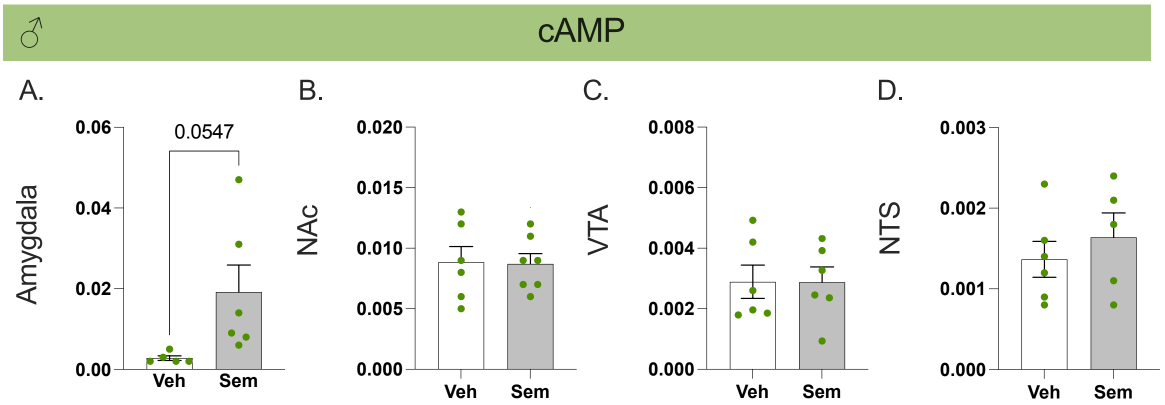

E.

Effects on cAMP after semaglutide treatment in alcohol naïve male mice		
Sex	*cAMP levels*	*Statistical analysis, unpaired t-test*
Males	amygdala	t(9)=2.21, P=0.0547
	NAc	t(11)=0.08, P=0.9383
	VTA	t(10)=0.02, P=0.9880
	NTS	t(9)=0.7449, P=0.4754

In comparison to vehicle, semaglutide (A) tends to increase the cAMP levels in amygdala, without effecting the cAMP levels in (B) NAc, (C) VTA or (D) NTS of alcohol naïve male mice. (E) Statistical summary of the presented data. Vehicle (Veh), semaglutide (Sem), Nucleus accumbens (NAc), ventral tegmental area (VTA), nucleus tractus solitarius (NTS).

Data are presented as mean ± SEM.

Supplementary Figure 15

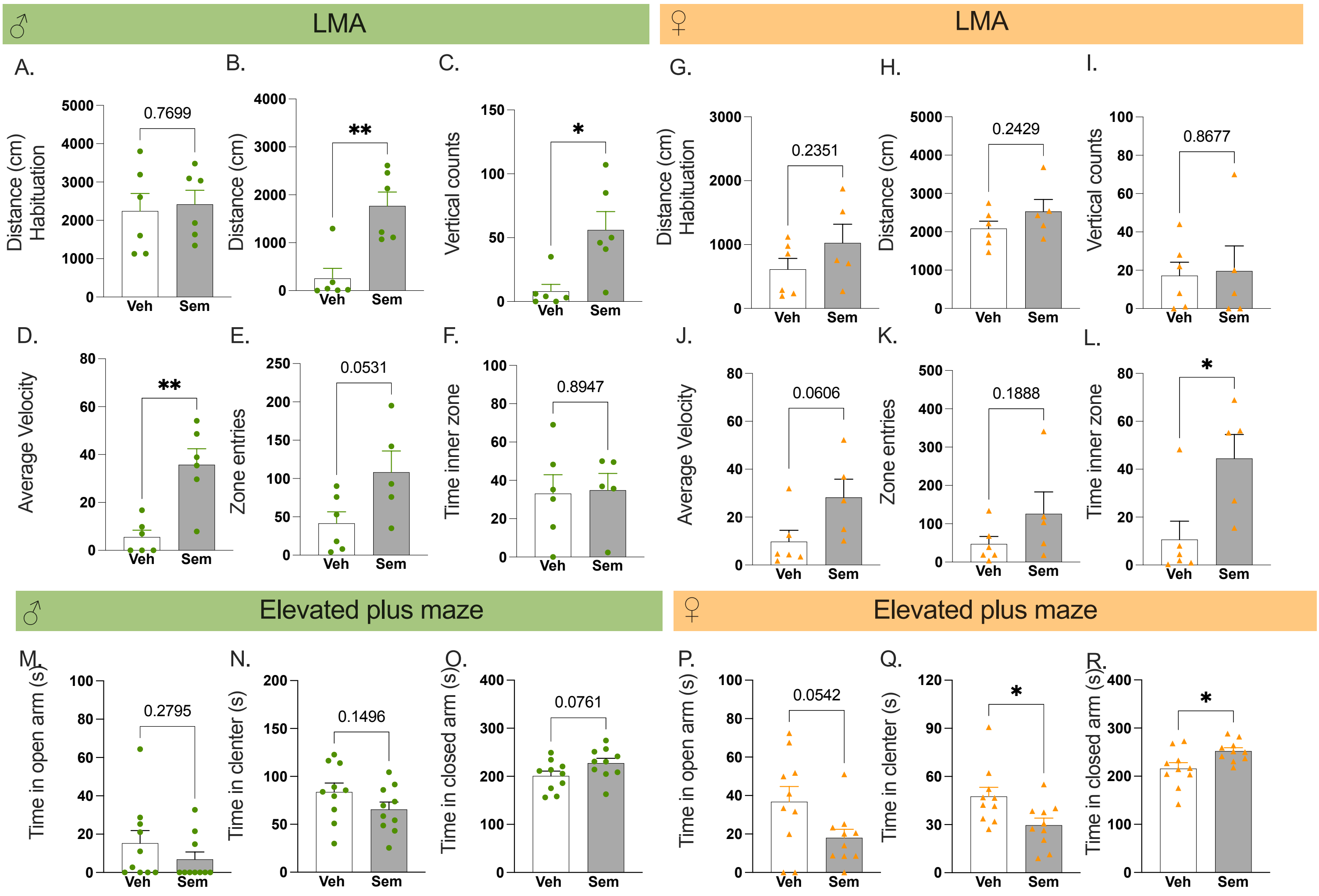

S.

Semaglutide effects on other behaviors in alcohol naïve male and female rats			
Sex	*Experiment*		*Statistical analysis, unpaired t-test*
Males	Locomotor activity	Distance, habituation	t(10)=0.30, P=0.7699
		Distance	t(10)=4.22, P=0.0018
		Vertical counts	t(10)=3.12, P=0.0109
		Average velocity	t(10)=4.18, P=0.0019
		Zone enteries	t(9)=2.23, P=0.0531
		Time in inner zone	t(9)=0.14, P=0.8947
Females		Distance, habituation	t(9)=1.27, P=0.2351
		Distance	t(9)=1.25, P=0.2429
		Vertical counts	t(9)=0.17, P=0.8677
		Average velocity	t(9)=2.15, P=0.0606
		Zone enteries	t(9)=1.42, P=0.1888
		Time in inner zone	t(9)=2.75, P=0.0226
Males	Elevated plus maze	Time in open arms	t(18)=1.12, P=0.2795
		Time in center	t(18)=1.51, P=0.1496
		Time in closed arms	t(18)=1.88, P=0.0761
Females		Time in open arms	t(18)=2.06, P=0.0542
		Time in center	t(18)=2.45, P=0.0247
		Time in closed arms	t(18)=2.58, P=0.0189

(A) In the locomotor activity test (LMA), the distance traveled during habituation is similar between male rats later treated with vehicle (Veh) or semaglutide (Sem). Semaglutide increases the (B) distance traveled, (C) vertical counts, (D) average velocity and (E) tends to increase zone entries, (D) without affecting time in inner zone. (G) The distance traveled during habituation is similar between female rats later treated with vehicle or semaglutide. Semaglutide treatment does not influence (H) distance, or (I) vertical counts. It tends to increase (J) average velocity without affecting (K) zone entries. Moreover, semaglutide (L) increases the time in inner zone. In the elevated plus maze test of male rats, semaglutide (M) does not influence time in open arm, (N) time in center, and (O) and tends to increase time in closed arms. In the elevated plus maze test of female rats, semaglutide (O) tends to decrease time in open arms, (Q) reduces the time in center, and (R) increases time in closed arms. (S) Statistical summary of the presented data.

Data are presented as mean ± SEM, significant data are illustrated by *P<0.05, **P<0.01.
