# Supplementary material for: Semaglutide reduces alcohol intake and relapse-like drinking in male and female rats
Source: eBioMedicine. 2023 Jun 7;93:104642. doi: 10.1016/j.ebiom.2023.104642 (PMC10363436; doi:10.1016/j.ebiom.2023.104642)
Supplement: Supplementary Tables S1 and S2 [file mmc2.docx]

**Supplementary Table 1.**

| Effects on noradrenaline and serotonergic neurotransmission after semaglutide treatment of alcohol drinking male or female rats | | | | | |
| --- | --- | --- | --- | --- | --- |
| *Sex* | *Brain area* | *Neurotransmitter* | *Vehicle*  *(Mean ±SEM)* | *Semaglutide*  *(Mean ±SEM)* | *Unpaired t-test* |
| Males | NAcS | NA | 3.4 ± 1.0 n=5 | 2.9 ± 0.5 n=5 | 0.6465 |
|  |  | 5-HT | 3.3 ± 0.5 n=5 | 3.1 ± 0.5 n=5 | 0.8547 |
|  |  | 5-HIAA | 1.8 ± 0.3 n=5 | 1.8 ± 0.2 n=5 | 0.9481 |
|  |  | 5-HIAA/5-HT | 0.6 ± 0.1 n=5 | 0.6 ± 0.1 n=5 | 0.5923 |
|  | NAcC | NA | 2.0 ± 0.5 n=5 | 2.1 ± 0.3 n=5 | 0.7892 |
|  |  | 5-HT | 0.8 ± 0.1 n=5 | 0.9 ± 0.1 n=5 | 0.7759 |
|  |  | 5-HIAA | 1.0 ± 0.1 n=5 | 1.3 ± 0.1 n=5 | 0.0670 |
|  |  | 5-HIAA/5-HT | 1.2 ± 0.1 n=5 | 1.6 ± 0.2 n=5 | 0.0853 |
|  | VTA | NA | 4.3 ± 0.4 n=5 | 5.4 ± 0.7 n=5 | 0.2061 |
|  |  | 5-HT | 1.6 ± 0.3 n=5 | 2.7 ± 0.4 n=5 | 0.0696 |
|  |  | 5-HIAA | 2.7 ± 0.5 n=5 | 3.5 ± 0.8 n=5 | 0.4350 |
|  |  | 5-HIAA/5-HT | 1.9 ± 0.3 n=5 | 1.3 ± 0.4 n=5 | 0.2886 |
| Females | NAcS | NA | 6.0 ± 0.1 n=5 | 5.8 ± 1.4 n=5 | 0.9358 |
|  |  | **5-HT** | **2.4 ± 0.2 n=5** | **3.5 ± 0.3 n=5** | **0.0164** |
|  |  | 5-HIAA | 0.9 ± 0.0 n=5 | 0.8 ± 0.1 n=5 | 0.1020 |
|  |  | 5-HIAA/5-HT | 4.1 ± 1.2 n=5 | 3.8 ± 0.6 n=5 | 0.1820 |
|  | NAcC | NA | 4.1 ± 1.2 n=5 | 3.8 ± 0.6 n=5 | 0.7813 |
|  |  | 5-HT | 0.9 ± 0.2 n=5 | 1.1 ± 0.2 n=5 | 0.3586 |
|  |  | 5-HIAA | 1.2 ± 0.2 n=5 | 1.6 ± 0.3 n=5 | 0.1878 |
|  |  | 5-HIAA/5-HT | 1.3 ± 0.1 n=5 | 1.5 ± 0.2 n=5 | 0.3629 |
|  | VTA | NA | 4.8 ± 1.5 n=5 | 5.5 ± 0.3 n=4 | 0.3600 |
|  |  | 5-HT | 2.2 ± 0.5 n=5 | 2.8 ± 0.6 n=4 | 0.4762 |
|  |  | 5-HIAA | 2.9 ± 0.4 n=5 | 4.2 ± 0.8 n=4 | 0.1698 |
|  |  | 5-HIAA/5-HT | 1.4 ± 0.2 n=5 | 1.6 ± 0.2 n=4 | 0.5068 |

Effects of semaglutide treatment on noradrenaline (NA), serotonin (5-HT), its metabolite 5-HIAA and turnover (5-HIAA/5-HT) in nucleus accumbens shell (NAcS), NAc core (NAcC), and the ventral tegmental area (VTA) of alcohol drinking male and female rats.

**Supplementary Table 2.**

| Effects on dopaminergic, noradrenalinergic and serotonergic neurotransmission after semaglutide treatment of alcohol drinking male or female rats | | | | | |
| --- | --- | --- | --- | --- | --- |
| *Sex* | *Brain area* | *Neurotransmitter* | *Vehicle*  *(Mean ±SEM)* | *Semaglutide*  *(Mean ±SEM)* | *Unpaired t-test* |
| Males | LDTg | DA | 0.12 ± 0.0 n=5 | 0.2 ± 0.1 n=5 | 0.0976 |
|  |  | **L-DOPA** | **1.8 ± 0.2 n=5** | **2.9 ± 0.4 n=5** | **0.0438** |
|  |  | DOPAC | 0.1 ± 0.0 n=5 | 0.2 ± 0.0 n=5 | 0.6877 |
|  |  | 3-MT | 0.7 ± 0.1 n=5 | 1.0 ± 0.2 n=5 | 0.2772 |
|  |  | HVA | 0.4 ± 0.1 n=5 | 0.5 ± 0.2 n=5 | 0.6311 |
|  |  | (DOPAC+HVA)/DA | 5.1 ± 1.6 n=5 | 3.8 ± 1.4 n=5 | 0.5426 |
|  |  | NA | 6.4 ± 0.4 n=5 | 9.6 ± 2.2 n=5 | 0.1867 |
|  |  | 5-HT | 2.3 ± 0.3 n=5 | 3.7 ± 0.5 n=5 | 0.0391 |
|  |  | 5-HIAA | 2.8 ± 0.3 n=5 | 4.5 ± 0.7 n=5 | 0.0543 |
|  |  | 5-HIAA/5-HT | 1.2 ± 0.1 n=5 | 1.2 ± 0.0 n=5 | 0.9177 |
|  | PVT | DA | 0.1 ± 0.0 n=5 | 0.1 ± 0.0 n=5 | 0.1536 |
|  |  | L-DOPA | 0.3 ± 0.1 n=5 | 0.2 ± 0.1 n=5 | 0.2433 |
|  |  | DOPAC | 0.3 ± 0.0 n=5 | 0.3 ± 0.1 n=5 | 0.5988 |
|  |  | 3-MT | 0.1 ± 0.0 n=5 | 0.1 ± 0.1 n=5 | 0.8060 |
|  |  | HVA | 0.2 ± 0.0 n=5 | 0.3 ± 0.1 n=5 | 0.6577 |
|  |  | (DOPAC+HVA)/DA | 6.0 ± 0.8 n=5 | 10.8 ± 2.6 n=5 | 0.1257 |
|  |  | NA | 5.4 ± 0.4 n=5 | 5.2 ± 0.6 n=5 | 0.8416 |
|  |  | 5-HT | 0.5 ± 0.1 n=5 | 0.4 ± 0.1 n=5 | 0.4779 |
|  |  | 5-HIAA | 1.9 ± 0.2 n=5 | 1.4 ± 0.3 n=5 | 0.1676 |
|  |  | 5-HIAA/5-HT | 4.4 ± 0.6 n=5 | 4.6 ± 0.8 n=5 | 0.8151 |
|  | LS | DA | 1.3 ± 0.1 n=5 | 1.2 ± 0.3 n=5 | 0.7934 |
|  |  | L-DOPA | 0.9 ± 0.1 n=5 | 0.9 ± 0.2 n=5 | 0.8087 |
|  |  | DOPAC | 0.5 ± 0.0 n=5 | 0.4 ± 0.1 n=5 | 0.2639 |
|  |  | 3-MT | 0.5 ± 0.0 n=5 | 0.6 ± 0.1 n=5 | 0.3457 |
|  |  | HVA | 0.4 ± 0.0 n=5 | 0.3 ± 0.1 n=5 | 0.2255 |
|  |  | (DOPAC+HVA)/DA | 0.7 ± 0.1 n=5 | 0.7 ± 0.1 n=5 | 0.8021 |
|  |  | NA | 5.1 ± 0.4 n=5 | 4.4 ± 0.5 n=5 | 0.2715 |
|  |  | 5-HT | 1.4 ± 0.1 n=5 | 1.2 ± 0.2 n=5 | 0.4848 |
|  |  | 5-HIAA | 1.3 ± 0.1 n=5 | 1.1 ± 0.2 n=5 | 0.2762 |
|  |  | 5-HIAA/5-HT | 1.0 ± 0.1 n=5 | 0.9 ± 0.1 n=5 | 0.7313 |
| Females | LDTg | DA | 0.1 ± 0.0 n=5 | 0.2 ± 0.0 n=4 | 0.1236 |
|  |  | L-DOPA | 1.5 ± 0.3 n=5 | 2.2 ± 0.2 n=4 | 0.1024 |
|  |  | DOPAC | 0.1 ± 0.0 n=5 | 0.2 ± 0.0 n=4 | 0.1879 |
|  |  | 3-MT | 0.3 ± 0.1 n=5 | 0.6 ± 0.0 n=4 | 0.0940 |
|  |  | HVA | 0.2 ± 0.0 n=5 | 0.3 ± 0.0 n=4 | 0.3761 |
|  |  | (DOPAC+HVA)/DA | 3.1 ± 0.6 n=5 | 2.1 ± 0.2 n=4 | 0.1927 |
|  |  | **NA** | **5.6 ± 0.5 n=5** | **7.6 ± 0.4 n=4** | **0.0250** |
|  |  | 5-HT | 1.8 ± 0.3 n=5 | 2.6 ± 0.2 n=4 | 0.0774 |
|  |  | 5-HIAA | 2.3 ± 0.5 n=5 | 3.2 ± 0.4 n=4 | 0.1997 |
|  |  | 5-HIAA/5-HT | 1.3 ± 0.1 n=5 | 1.2 ± 0.0 n=4 | 0.4438 |
|  | PVT | DA | 0.1 ± 0.0 n=5 | 0.1 ± 0.0 n=5 | 0.8246 |
|  |  | L-DOPA | 0.4 ± 0.1 n=5 | 0.7 ± 0.3 n=5 | 0.4424 |
|  |  | DOPAC | 0.4 ± 0.1 n=5 | 0.4 ± 0.0 n=5 | 0.7803 |
|  |  | 3-MT | 0.2 ± 0.0 n=5 | 0.3 ± 0.1 n=5 | 0.1762 |
|  |  | **HVA** | **0.2 ± 0.0 n=5** | **0.3 ± 0.0 n=5** | **0.0101** |
|  |  | (DOPAC+HVA)/DA | 6.1 ± 0.7 n=5 | 9.2 ± 1.3 n=5 | 0.0730 |
|  |  | NA | 6.8 ± 0.6 n=5 | 7.9 ± 0.6 n=5 | 0.2326 |
|  |  | 5-HT | 0.6 ± 0.1 n=5 | 0.9 ± 0.3 n=5 | 0.4036 |
|  |  | 5-HIAA | 2.0 ± 0.1 n=5 | 2.6 ± 0.4 n=5 | 0.1996 |
|  |  | 5-HIAA/5-HT | 3.7 ± 0.4 n=5 | 3.6 ± 0.5 n=5 | 0.9781 |
|  | LS | DA | 1.2 ± 0.2 n=5 | 1.4 ± 0.2 n=5 | 0.3954 |
|  |  | L-DOPA | 0.4 ± 0.1 n=5 | 0.7 ± 0.3 n=5 | 0.2191 |
|  |  | DOPAC | 0.6 ± 0.1 n=5 | 0.6 ± 0.3 n=5 | 0.9641 |
|  |  | 3-MT | 0.3 ± 0.0 n=5 | 0.5 ± 0.1 n=5 | 0.1433 |
|  |  | HVA | 0.5 ± 0.1 n=5 | 0.3 ± 0.0 n=5 | 0.2475 |
|  |  | (DOPAC+HVA)/DA | 0.9 ± 0.1 n=5 | 0.7 ± 0.1 n=5 | 0.0746 |
|  |  | NA | 6.0 ± 1.1 n=5 | 5.9 ± 0.6 n=5 | 0.9863 |
|  |  | 5-HT | 1.2 ± 0.2 n=5 | 1.7 ± 0.3 n=5 | 0.1965 |
|  |  | 5-HIAA | 1.4 ± 0.2 n=5 | 1.7 ± 0.3 n=5 | 0.4565 |
|  |  | 5-HIAA/5-HT | 1.2 ± 0.0 n=5 | 1.0 ± 0.0 n=5 | 0.0592 |

Effects of semaglutide treatment on dopamine (DA), its precursor (L-DOPA) and metabolites (DOPAC, 3-MT, HVA), turnover ((DOPAC+HVA)/DA), noradrenaline (NA), serotonin (5-HT), its metabolite 5-HIAA and turnover (5-HIAA/5-HT) in laterodorsal tegmental area (LDTg), paraventricular thalamus (PVT), lateral septum (LS) of alcohol drinking male and female rats.
